# Can We Boost *N*-Glycopeptide Identification Confidence? Smart Collision Energy Choice Taking into Account Structure and Search Engine

**DOI:** 10.1021/jasms.3c00375

**Published:** 2024-01-29

**Authors:** Helga Hevér, Andrea Xue, Kinga Nagy, Kinga Komka, Károly Vékey, László Drahos, Ágnes Révész

**Affiliations:** † MS Proteomics Research Group, HUN-REN Research Centre for Natural Sciences, Magyar Tudósok körútja 2., Budapest H-1117, Hungary; ‡ Faculty of Science, Institute of Chemistry, Hevesy György PhD School of Chemistry, Eötvös Loránd University, Pázmány Péter sétány 1/A, Budapest H-1117, Hungary; § Department of Chemical and Environmental Process Engineering, Budapest University of Technology and Economics, Budapest H-1111, Hungary

**Keywords:** tandem mass spectrometry, bottom-up proteomics, *N*-glycosylation, glycan structure, identification score, search engine, collision energy optimization, general linear model, lasso regression

## Abstract

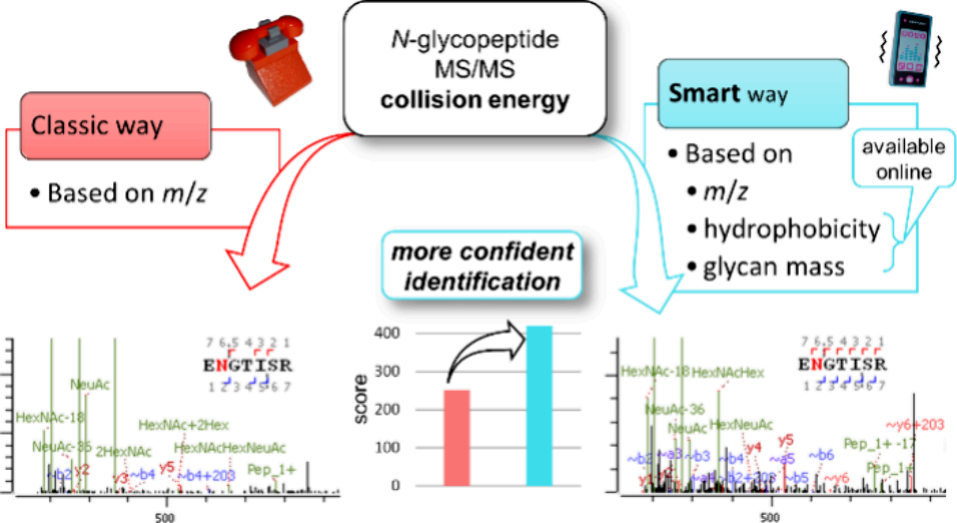

High confidence and reproducibility are still challenges in bottom-up mass spectrometric *N*-glycopeptide identification. The collision energy used in the MS/MS measurements and the database search engine used to identify the species are perhaps the two most decisive factors. We investigated how the structural features of *N*-glycopeptides and the choice of the search engine influence the optimal collision energy, delivering the highest identification confidence. We carried out LC-MS/MS measurements using a series of collision energies on a large set of *N*-glycopeptides with both the glycan and peptide part varied and studied the behavior of Byonic, pGlyco, and GlycoQuest scores. We found that search engines show a range of behavior between peptide-centric and glycan-centric, which manifests itself already in the dependence of optimal collision energy on *m*/*z*. Using classical statistical and machine learning methods, we revealed that peptide hydrophobicity, glycan and peptide masses, and the number of mobile protons also have significant and search-engine-dependent influence, as opposed to a series of other parameters we probed. We envisioned an MS/MS workflow making a smart collision energy choice based on online available features such as the hydrophobicity (described by retention time) and glycan mass (potentially available from a scout MS/MS). Our assessment suggests that this workflow can lead to a significant gain (up to 100%) in the identification confidence, particularly for low-scoring hits close to the filtering limit, which has the potential to enhance reproducibility of *N*-glycopeptide analyses. Data are available via MassIVE (MSV000093110).

## Introduction

Glycosylation is one of the most common post-translational modifications (PTMs) of proteins and the resulting glycoproteins are key regulating molecules in many biological processes and cellular events.^1^ Further, they can serve as biomarkers since aberrant glycosylation pattern is associated with the progression of various diseases^2−4^ and several protein therapeutics are also glycosylated.^5^ Their analytical screening is often performed using mass spectrometry (MS) coupled to liquid chromatography (LC or nano-LC) techniques.^6−8^ Glycosylation is most commonly mapped through the LC-MS/MS investigation of intact *N*-glycopeptides obtained from proteolytic cleavage (most frequently, trypsin is used). Almost exclusively in a data dependent analysis (DDA) setup, this approach allows the determination of modification site, the identification of carrying peptides and provides information on structure and composition of the glycan attached.^3,9−12^

Various fragmentation techniques have already been explored in LC-MS/MS workflows, and they may provide somewhat complementary information.^3,10,13−18^ Beam type collision-induced dissociation (CID) enables rapid MS/MS scanning, is widely available, and has the ability to access both glycan and peptide fragments. It can be implemented on both quadrupole time-of-flight (QTof) and Orbitrap instruments (in this case, it is also called higher-energy collisional dissociation, HCD).^19,20^ Electron-based techniques (electron transfer dissociation, ETD, and electron capture dissociation, ECD) are outstanding in site-localization, but this is more relevant for O-glycosylation lacking the consensus sequence.^21−24^ Combinations have also been implemented, namely, electron transfer/higher-collisional dissociation (EThcD) and electron transfer/collision induced dissociation (ETciD).^7,23^ Systematic comparison of the various fragmentation modes combined with several experimental parameters revealed that collision induced dissociation with well-chosen settings is outstanding with regard of number of identifications for *N*-glycopeptide analysis.^23^ Therefore, CID/HCD is the preferred MS/MS technique for *N*-glycopeptide analysis.

The *N*-glycopeptides are typically identified through a database search. The measured MS/MS spectra are searched against the potential *N*-glycopeptide candidates constructed from the potential protein sequences (protein database) and glycan structures (glycan database). In some rare cases, de novo glycan sequencing is applied. The quality of the determined peptide-spectrum-matches are characterized by a search engine specific score value.^3,6,14,25^ We note that despite the various developments of the last few decades in both LC-MS/MS instrumentation and identification bioinformatics tools, the *N*-glycopeptide identification is still far from trivial due to experimental and data handling issues;^10,12,26^ for example, a recent study showed significant team-to-team variations in the *N*-glycopeptide identifications even when the same MS/MS data were evaluated.^26^

The feasibility of identifying an *N*-glycopeptide from a CID/HCD MS/MS spectrum depends on the information content of the spectrum, which is, in turn, highly influenced by the collision energy (CE) value applied. It was found that low CE breaks the more fragile glycan structure, providing data about the oligosaccharide connectivity, whereas larger CE results in b- and y-type ions from the cleavages of the peptide backbone.^3,14,27−35^ As a result, the most widespread solution is the combination of low- and high-energy spectra of the same precursor (stepped collision energy–sce method).^3,23,27−32,36−38^ Further, the chosen CE is typically linearly dependent on the size (characterized by *m*/*z*) of the *N*-glycopeptide,^28,38^ which is embedded in the definition of the NCE% (normalized collision energy) term of Orbitrap instruments.^39^

Several works addressed the determination of the optimal linear fit/NCE% values based on the energy dependent investigation of *N*-glycopeptide fragmentation.^23,27,28,31,38,40^ It was found that optimal setting may be mass spectrometer dependent even when a different member of the same instrument series is used.^40^ Since peak picking and scoring system of the glycopeptide software solutions may be significantly different, optimal experimental parameters may be also influenced by the data analysis workflow applied which phenomenon was proved for unmodified peptides.^41^ We note in passing that the CE setting can alternatively be optimized also to detect fragmentation pattern diagnostic for specific glycans.^34,37,42,43^

Regarding the generic workflows, it has been observed that linear optimal CE vs *m*/*z* correlation still has large residuals.^38^ Some relevant factors have been identified in the literature but always on a limited set of glycopeptides and/or from the view of fragmentation (not from scoring/confident identification). Furthermore, the findings are somewhat contradictory to each other. Several studies showed that amino acid composition influences the fragmentation of positively charged *N*-glycopeptides.^27,38,44^ Even dissociation of deprotonated *N*-glycopeptides is affected by peptide sequence, although in that project peptide length was found dominating which can be explained by degrees of freedom effect.^45^ On the other hand, another research work claimed that no or just negligible influence of peptide sequence exists which may be due to few *N*-glycopeptides investigated.^28^ Charge dependence also has ambiguity based on the literature: analysis of tryptic digest of glycoprotein standards showed that activation energy decreases with increasing charge state.^35^ Two other studies found no influence of charge,^27,28^ one of them applied Orbitrap instrument therefore charge is likely to hidden in NCE%.^27^ Discrepancy may be due to a limited number of species and/or variation in proton mobility at the same time. It was found that precursor proton mobility predicts CE needed for glycan fragmentation but peptide fragmentation is unrelated.^33,46^ Fragmentation behavior appeared to be not affected by the size, composition and type (i.e., high mannose or hybrid) of attached oligosaccharide structure.^27,44,46^ Finally, charge carrier was found to be relevant which is associated with the different location of the attached Na^+^ and H^+^.^47,48^ In summary, all these results point to the need of a more systematic study of factors influencing the optimal energetics of fragmentation on a diverse set of glycopeptides.

In the present project, we carried out systematic collision energy resolved mass spectrometric experiments using a single CE setting to obtain a data set of glycopeptide-level optimal CEs in terms of score with various peptide sequences and glycan structures–few hundred of *N*-glycopeptides were investigated. We aimed (1) to identify and confirm qualitative trends in glycopeptide optimal CE as a function of structure for multiple search engines and to understand what matters beyond *m*/*z*; (2) to reveal which of these characteristics may be used to enhance *N*-glycopeptide identification performance, e.g., in an online setup; and (3) to perform evaluation for 3 search engines to highlight differences that can lead to different interpretation of data. Over the years, dozens of software solutions have been developed for intact glycopeptide analysis.^49^ In the present work, search engines with different search strategies (peptide-first and glycan-first) were applied, and they were also selected based on their availability and compatibility with our LC-MS/MS data. Comparison involved the most frequently used commercial Byonic search engine^50^ and the freely available pGlyco.^51^ Additionally, the GlycoQuest search engine scoring only the glycan part was applied.

## Experimental Section

### Glycoprotein Standards

Three glycoprotein standards (AGP, fetuin, and transferrin) were used for the investigations after enzymatic digestion to a mixture of peptides and *N*-glycopeptides. Our standard laboratory protocol was applied, based on denaturation with Rapigest SF, reduction by dithiothreitol, alkylation using iodoacetamide, and digesting first by Lys-C/trypsin, then by trypsin in an ammonium bicarbonate buffer. After quenching and drying, aliquots of 200 pmol were dissolved in injection solvent (98% water, 2% acetonitrile, and 0.1% formic acid) prior to the nano-LC-MS/MS analysis (see Material S1). A mixture of three glycoprotein standards was also prepared from the digests of AGP, fetuin and transferrin.

### Complex Samples

*N*-Glycopeptides obtained from complex samples were also studied: blood plasma and HeLa tryptic digests were used. Blood plasma was digested in aliquots of 30 μg according to the same protocol as the glycoprotein standards. The blood plasma digest was dried in SpeedVac and cleanup was performed using C_18_ spin column (Thermo Fisher Scientific) in aliquots of 15 μg using a protocol based on manufacturer’s recommendation. The resulting samples were again dried in SpeedVac. Then both HeLa tryptic digest and blood plasma digest were subject to a simple and cheap acetone solvent precipitation method in aliquots of 1 μg (see Material S2).^52,53^ This procedure is a rapid and highly selective glycopeptide enrichment method, based on the difference in solubility of glycosylated and nonglycosylated peptides in acetone.

### Mass Spectrometric Measurements

Nano-LC-MS/MS studies of the digested glycoprotein standards and complex protein samples enriched in glycopeptides were performed using our standard laboratory methods for glycoproteomics investigation with varying single MS/MS collision energy settings. Samples were subjected to nano-LC-MS/MS analysis using a Dionex UltiMate 3000 RSLCnano LC system coupled to a Bruker Maxis II ETD Q-TOF via a CaptiveSpray nanoBooster ionization source operated in positive mode. Sample digest was injected onto an Acclaim PepMap 100 C18 trap column (5 μm, 100 Å, 100 μm × 20 mm, Thermo Fisher Scientific, Waltham, MA, USA) using 0.1% trifluoroacetic acid (TFA). (Glyco)peptides were separated on an Acquity M-Class BEH130 C18 analytical column (1.7 μm, 130 Å, 75 μm x 250 mm Waters, Milford, MA) at 48 °C using a flow rate of 300 nL/min. The gradient was as follows: 4% B from 0 to 11 min, followed by a 120 min gradient to 50% B, and then the concentration of the solvent B was elevated to 90% in 1 min and kept there for 10 min; solvent A was 0.1% formic acid (FA) in water, while solvent B was 0.1% FA in acetonitrile.

Sample ionization was achieved in positive electrospray ionization mode via a CaptiveSpray nanoBooster ion source using acetonitrile as the solvent in the nanoBooster. The capillary voltage was set to 1300 V, the nanoBooster pressure was 0.2 bar, the drying gas was heated to 150 °C, and the flow rate was 3 L/min.

Spectra were collected using a fixed cycle time of 2.5 s and the following scan speeds: MS spectra at 2 Hz, CID on precursors at 4 Hz for abundant ones, and at 0.5 Hz for peaks of low abundance. An active exclusion of 2 min after 1 spectrum was used except if the intensity of the precursor was elevated 3-fold. The use of exclusion is typical in mass spectrometry based proteomics measurements; our respective settings are the typical values of Bruker instruments.

In order to take into account the size of the species investigated, the CE linearly dependent on *m*/*z* was employed in all of our experiments. Our energy dependent measurement series involved 27 nano-LC-MS/MS runs with different settings. Our starting point for optimization referred to as 100% was 50 eV at *m*/*z* 600 and 135 eV at *m*/*z* 2000 with a linear interpolation between the two *m*/*z* values. This setting equals to the high CE component of the method published by Hinneburg et al.^28^ Then, the CE was systematically varied from 6.25 to 175% in steps of 6.25%. The largest value that can be set at our instrument is 200 eV; therefore, an upper limit was used at this value. We deliberately used a single CE setting throughout the measurements so that CE optimal for peptide and glycan fragmentation could be investigated separately. Experiments were performed with the use of inclusion lists based on DDA measurements taken with sce method optimized for our mass spectrometric platform.^38^ Altogether four lists were created: two lists for the mixture of the 3 glycoprotein standards and one list for both HeLa and blood plasma samples.

The reason for the use of inclusion lists were 2-fold: to ensure that a given *N*-glycopeptide is selected for MS/MS measurement at all CE settings resulting in better energy dependent score curves and to ensure careful, balanced, representative selection of glycopeptide compounds for analysis. It is important to keep in mind that there should be diversity in terms of (1) glycan structure (e.g., different high-mannose types, complex multiantennary glycans, various sialylation levels, fucose variants), (2) peptide properties (e.g., amino acid sequence, length, hydrophobicity), (3) different charge levels, and (4) wide retention range. These requirements can be better fulfilled applying preplanned *N*-glycopeptide lists. In our work, we aimed to extend the method to as wide range of structures as possible so that we have a diverse data set of CE values with good accuracy, and the developed procedure can be applied to “any” *N*-glycosylated sample with a good approximation. The complete inclusion lists of *N*-glycopeptides (together with peptide sequence, glycan composition, charge, *m*/*z*, accession number and protein ID data) can be found in SI (see Tables S1–S4). Furthermore, Figures S1 and S2 depict the high diversity of the studied species. Figure S2 also highlights that the “HexNAc(4)Hex(5)NeuAc(2)” doubly sialylated biantennary glycoform was measured with diverse peptide sequences and different charge levels through the recording of 44 measurement points. In addition, Figure S1 shows that the peptide with the amino acid sequence “QDQCIYNTTYLNVQR” was mapped with 34 different measurement points, which represent different glycan structures and different charge levels during the measurement of different samples.

The variability of peptides can be considered almost “unlimited”, but *N*-glycoforms still appear in nature with limited, defined structures, so in the case of mapping *N*-glycopeptides, a target range can be outlined. The presented *N*-glycopeptide list was therefore intended to display this wide, complex range as authentically as possible in order to collect reliable data during studies aimed at determining the optimal CE value of *N*-glycopeptides.

### Data Analysis

#### N-Glycopeptide Identification Using Various Search Engines

The raw QTof data were first recalibrated using Bruker Compass DataAnalysis software v 4.3 (Bruker Daltonik GmbH, Bremen, Germany) for the internal calibrant. MS/MS spectra were searched against the appropriate protein and/or glycan database using Byonic v3.8.13 (Protein Metrics, Cupertino, CA),^50^ pGlyco 2.0^51^ and GlycoQuest search engines (see Material S3). *N*-glycopeptide identifications were accepted with the following filtering criteria based on literature suggestions and personal experience:^28,50,54^ Byonic score > 300 or pGlyco total score > 15 or GlycoQuest score > 30. The Byonic Excel reports, pGlyco FDR-Pro.txt reports, and GlycoQuest Excel reports obtained from ProteinScape were input files for data aggregation carried out by Serac program^55^ in the energy dependent studies used for energy dependence investigations (see Determination of Optimal CE Setting Using Serac).

#### Determination of Optimal CE Setting Using Serac

For the determination of optimal CE of the large number of *N*-glycopeptides for the various identification scores, we used our recently developed program called Serac.^55^ The program collected the score values (i.e., Byonic score, pGlyco peptide score, pGlyco glycan score, pGlyco total score, and GlycoQuest score) as a function of collision energy from the energy-dependent mass spectrometric data series and determined the optimal CE as the center of a Gaussian function fitted to the data points. To ensure that we draw conclusions based on confident *N*-glycopeptide identifications, we only selected species meeting certain requirements regarding minimum scores and number of consecutive CE values at which they were identified. The nonlinear fits were carried out by Serac, and the corresponding plots were generated using the levmar^56^ and PGPLOT^57^ libraries through their Perl Data Language interfaces (see Material S4). The optimal CE data of the five different scores were subjected to statistical analysis.

#### General Linear Model Statistical Analysis

For structural properties that were deemed relevant by our judgment, we carried out ANOVA/ANCOVA-type analyses, formulated as general linear models, with the help of the Statistica program package. We investigated whether the chosen categorical (e.g., charge) or continuous (e.g., hydrophobicity) variables bear statistical significance besides *m*/*z* in describing the optimum collision energy. We carried out separate tests on the homogeneity of slopes and applied models accordingly (Table S5).

#### Machine Learning Model: Lasso Regression

To identify potential further glycopeptide characteristics that could be relevant in determining the optimal CE in a way “unbiased” by human judgment, we employed lasso regression. Lasso is a variant of linear regression where a penalty term is included that considers the size of the slopes. Increasing the coefficient of this penalty term results in more and more zero slopes, leading to an efficient selection of good explanatory variables from a potentially linearly dependent set.

We regressed the optimal collision energy against a large set of glycopeptide parameters, including amino acid and glycan composition, peptide and glycan masses, charge, etc. (see the list in Material S5). We varied the regularization coefficient in an empirical way to find a minimum set of parameters above which no significant increase of *R*^2^ or decrease of the mean-squared error was observable. We employed the sklearn python package for this purpose.

## Results and Discussion

### N-Glycopeptide Level Optimal Collision Energy

In line with earlier studies of ours, in order to get insight into the behavior of individual species, we measured how the various search engine scores depend on collision energy and identified the best performing single CE for each *N*-glycopeptide in our data set. We would like to emphasize that single CE was used; using stepped CE typical for glycopeptide analysis would have impaired our ability to differentiate between search engine scores and effects related to the peptide and glycan parts.

Experiments were carried out on the mixture of the tryptic digests of the three glycoprotein standards, on HeLa tryptic digest enriched in *N*-glycopeptides, and on blood plasma tryptic digest enriched in *N*-glycopeptides. To ensure that a given *N*-glycopeptide is selected for MS/MS measurement at all CE settings and thereby identified at as many CE settings as possible, inclusion lists were determined from preceding DDA experiments. Our starting setting (the “100%”) was the high CE component of Hinneburg et al.^28^ and then we increased and decreased the CE value in the steps of 6.25%. Overall, we applied 27 nano-LC-MS/MS methods mapping the CE range from 6.25 to 175%.

Then, we constructed CE dependence curves of several identification scores for *N*-glycopeptides, namely, Byonic score, pGlyco peptide score, pGlyco glycan score, pGlyco total score, and GlycoQuest score. Byonic is the most widely used search engine for glycosylation and is known to be more focused on the peptide backbone of the glycopeptide; the attached glycan is handled as a variable modification. GlycoQuest annotates glycan fragments; therefore, it provides information on the oligosaccharide structure. The peptide part is only characterized by its total mass. Finally, pGlyco gives separate scores for glycan and peptide identification. When a given species was identified more than once in the same LC-MS/MS run, that is, measured several times at the same CE setting, the best scoring match was accepted.

The optimal CE value depends not only on the structure but also on the charge state; therefore, *N*-glycopeptides at different charge states were tested where possible. In order to allow for a differential investigation of the optimal CE properties associated with the different charge states of a single *N*-glycopeptide, these species were treated as separate data points in this study. Overall, we could identify 280, 242, and 244 *N*-glycopeptide species from all nano-LC-MS/MS measurements using Byonic (score > 300), pGlyco (total score > 15) and GlycoQuest (score > 30) search engines, respectively. Among these, 270 (Byonic score), 104 (pGlyco peptide score), 130 (pGlyco glycan score), 96 (pGlyco total score), and 190 (GlycoQuest score) *N*-glycopeptide species fulfilled the requirements for the energy-dependent investigation (see Data Analysis). Merging the different charge states, the number of *N*-glycopeptide structures found by each search engine was as follows: Byonic 169, pGlyco peptide 76, pGlyco glycan 92 and Glycoquest 128. Combining the results of the four search engines, the total number of unique *N*-glycopeptide structures examined in the energy-dependent tests was 184, comprising 70 different peptide backbones and 39 different oligosaccharide structures (see Table 1).

**Table 1:** Number of Identified *N*-Glycopeptides Species, Number of Different Peptide Sequences, and Number of Different Glycans for the Various Search Engines^a^

	Byonic	pGlyco peptide	pGlyco glycan	pGlyco total	Glyco-Quest	Combined number from the four search engine results^c^
# of *N*-glycopeptide species identified from all runs	280		242		244	
# of *N*-glycopeptide species considered in CE study	270	104	130	96	190	465
# of *N*-glycopeptide structures considered in CE study	169	76	92		128	184
# of different peptide sequences	58	28	33	26	56^b^	70
# of different glycans	38	24	27	24	26	39

a Results are combined for the standards, HeLa, and blood plasma samples.

b GlycoQuest search engine reported peptide masses which were correlated to peptide sequences identified by Byonic and/or pGlyco.

c Without the repeated appearance of a component.

**Figure 1:**
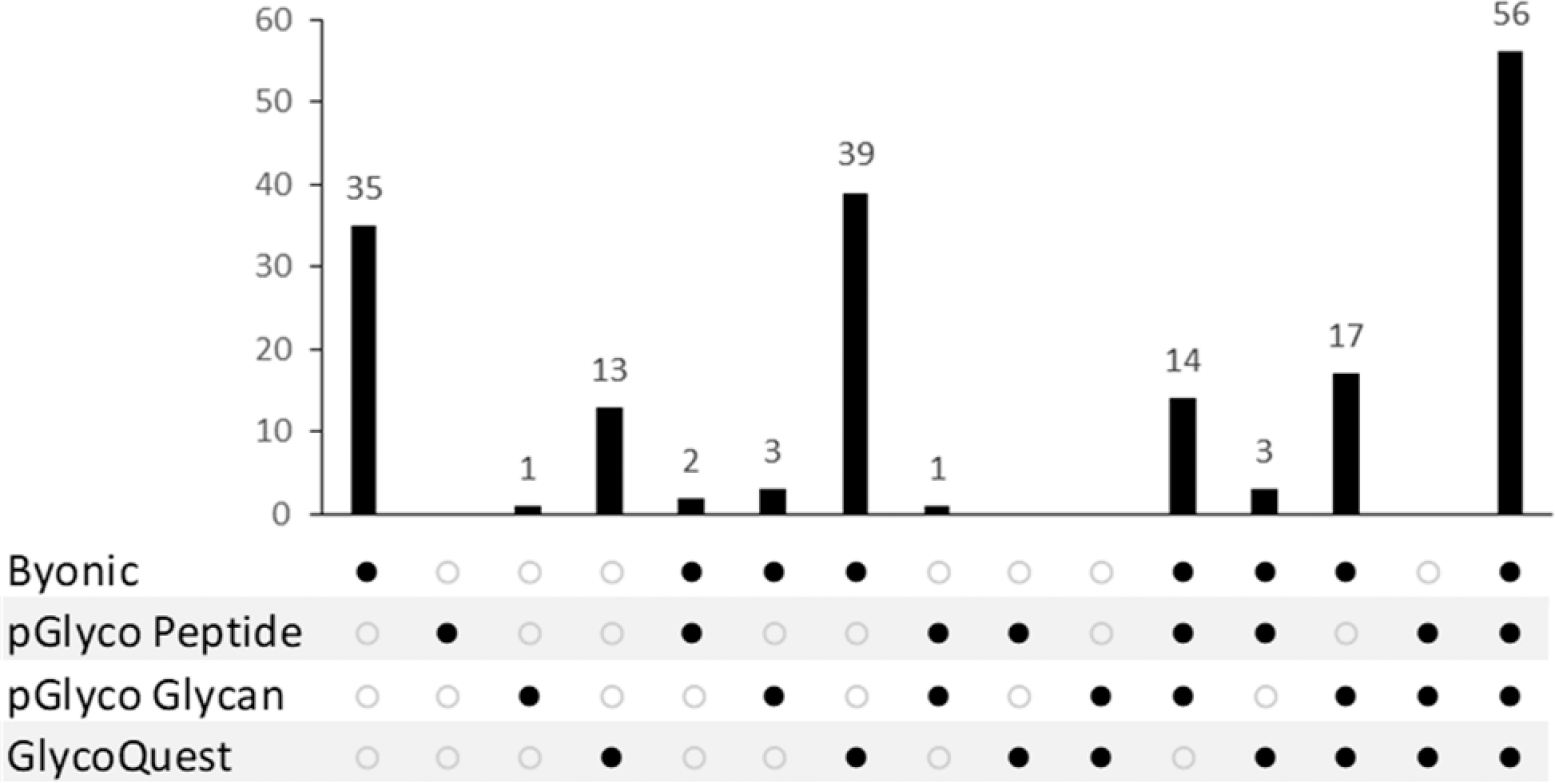
Upset plot of the hit distribution of 184 unique *N*-glycopeptide structures based on the evaluations of the four search engines.

Comparing the search engine results, all four engines found 56 structures out of 184 *N*-glycopeptide structures (Figure 1). Although the joint hit rate of the software pair Byonic-pGlyco (39) was still outstanding, the Byonic search engine alone had 35 unique *N*-glycopeptide hits. Lists of the *N*-glycopeptide structures corresponding to the upset plot can be found in the SI (Figure S3).

Representative examples of experimental points together with the fitted Gaussian functions for all investigated identification scores are depicted in Figure S4 for example, peptide QDQCIYNTTYLNVQR-HexNAc(5)Hex(6)NeuAc(2)^4+^ (derived from AGP, ID: P02763 · A1AG1_HUMAN). The results clearly show that the optimal CE of the two glycan scores, namely GlycoQuest and pGlyco glycan, are close to each other. Furthermore, they are ca. half of the optimal CE of pGlyco peptide score. Byonic score optimum lies next to pGlyco peptide optimum. Finally, pGlyco total is in between the two separate pGlyco scores.

### Starting Point: Search Engine Dependence and Comparison

**Figure 2:**
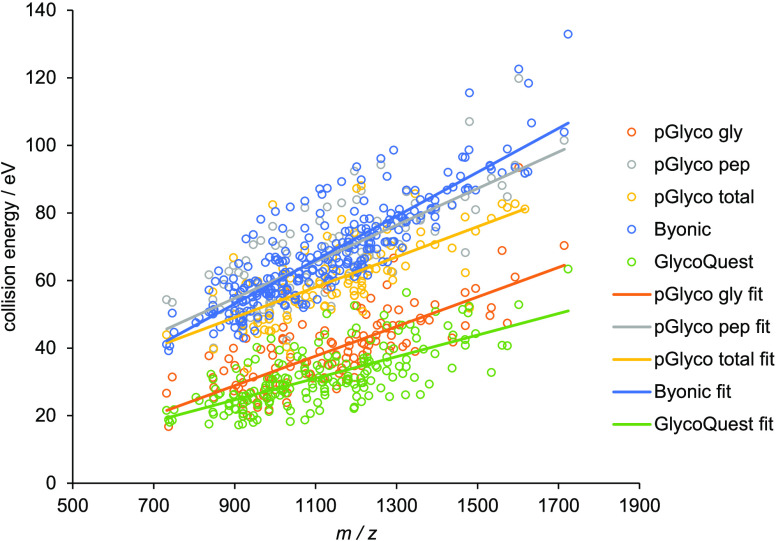
Optimal collision energies of *N*-glycopeptides in eV as a function of *m*/*z* for the various search engine scores. Circles indicate the experimental points, while lines represent linear fits of the measured data. Blue: Byonic; gray: pGlyco peptide; yellow: pGlyco total; orange: pGlyco glycan; green: GlycoQuest.

As a next step, we plotted the optimal CE values of *N*-glycopeptides as a function of *m*/*z* for all of the investigated *N*-glycopeptide species. From earlier works,^38,40^ we obviously expected a linear *m*/*z* dependence of optimum CEs. Figure 2 shows that this is indeed the case: all investigated identification scores follow the anticipated trend.

Significant differences between the various scores can be observed, matching the behavior of the representative example glycopeptide. The two scores belonging to the glycan parts (i.e., pGlyco glycan and GlycoQuest) run next to each other. Fragmentation of the glycan requires about half of the energy compared to the fragmentation of peptide backbone (e.g., pGlyco peptide score), in line with the literature.^27,28,30^ Then, Byonic trendline lies very close to pGlyco peptide, at large *m*/*z* values at even higher CE, corroborating the peptide-centric nature of Byonic.^26^ As expected, pGlyco total combining the characterization of peptide and glycan fragmentation goes between the pGlyco peptide and pGlyco glycan. Therefore, we will not discuss the behavior of the pGlyco total score in detail during further analysis.

### Impact of Individual Structural Properties on Trends

Having the global trendline at hand (Figure 2), we turned to the impact of individual properties on the optimum CE for the various investigated scores. To do so, we formed subgroups from the data points according to several properties and checked if individual trendlines fitted to them differ. As there is a general *m*/*z* dependent trend in the collision energies, we used a general linear model (GLM) to assess which characteristics have an impact on the slope and/or intercept of the *m*/*z* dependence of CE.

#### Sequence

One of the influencing factor in some cases is peptide sequence, which was already implied for Byonic score using sce methods.^38^ Here, we found the largest impact of sequence for the Byonic score and pGlyco peptide score. For example, the outlier ENGTISR (derived from AGP, ID: P02763 · A1AG1_HUMAN) has well-separated sets of points with different slopes and intercepts. The total score of pGlyco behaves similarly. In contrast, scores characterizing the glycan fragmentation showed small (pGlyco glycan, GlycoQuest) separations between sequences; even ENGTISR follows similar trend to the other *N*-glycopeptides. Oligosaccharide fragmentation thus appears to be much less influenced by the peptide sequence. Therefore, when the goal is targeted analysis of a certain peptide (e.g., a certain glycosylation site), a specific CE setting optimized to the given amino acid sequence may be useful.

**Figure 3:**
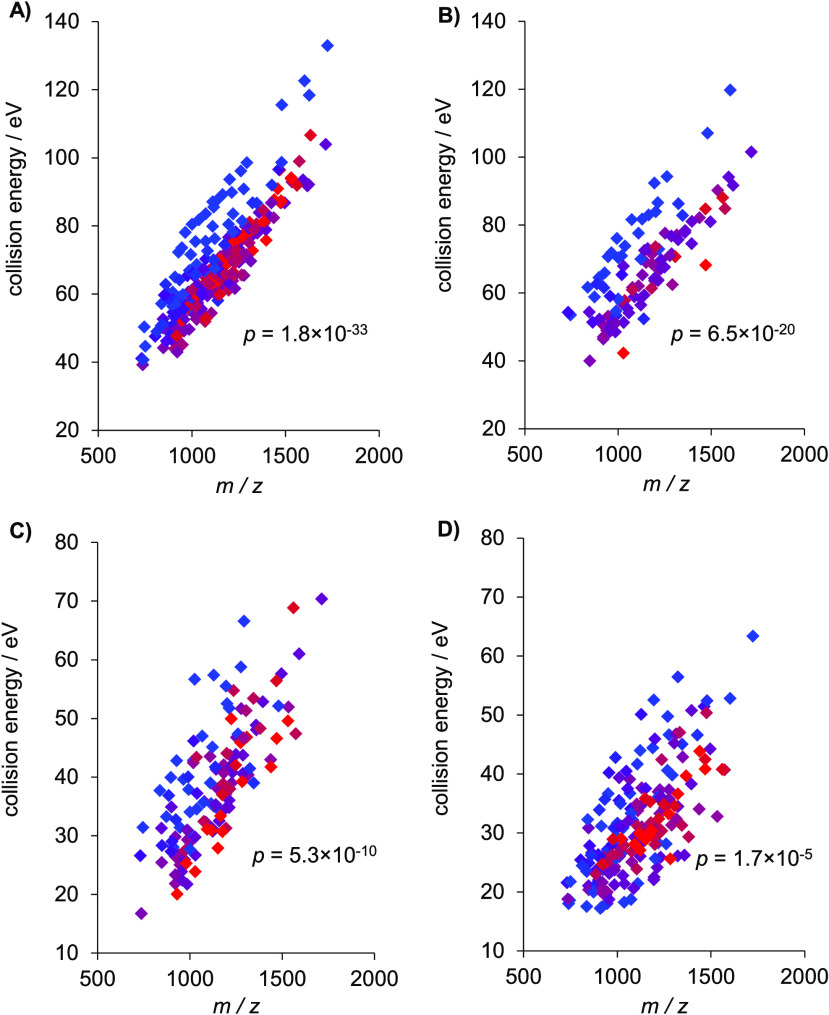
Optimal collision energies of *N*-glycopeptides in eV as a function of *m*/*z* for the various search engine scores. (A) Byonic, (B) pGlyco peptide, (C) pGlyco glycan, and (D) GlycoQuest. Colors of data points indicate the hydrophobicity of the peptide backbone: range goes from blue to red belonging to the lowest and largest value, respectively. The *p* values show statistical significance of hydrophobicity.

Since the peptide backbone is not known before identification, we investigated if we could utilize certain quantitative properties to characterize it. We found that peptide hydrophobicity is a good numerical representation because it is well correlated with the chromatographic retention time (see Figure S5), therefore is a promising characteristic that might be used in real measurement workflow. We determined hydrophobicity values using the peptide analyzing tool of Thermo Fisher Scientific.^58^ It can be seen in Figure 3 that optimal CEs for all investigated identification scores show statistically significant dependence on hydrophobicity, but the behavior of search engines is different: while optimal CE of Byonic and pGlyco peptide scores are highly affected, the pGlyco glycan score and especially the GlycoQuest score are much less influenced. Our present study on a few hundred of *N*-glycopeptides obviously highlights the influence of amino acid sequence on the fragmentation of peptide backbone, therefore, earlier work finding no peptide backbone dependence most likely used too few species to draw precise conclusions in this respect.^28^

#### Number of Mobile Protons

**Figure 4:**
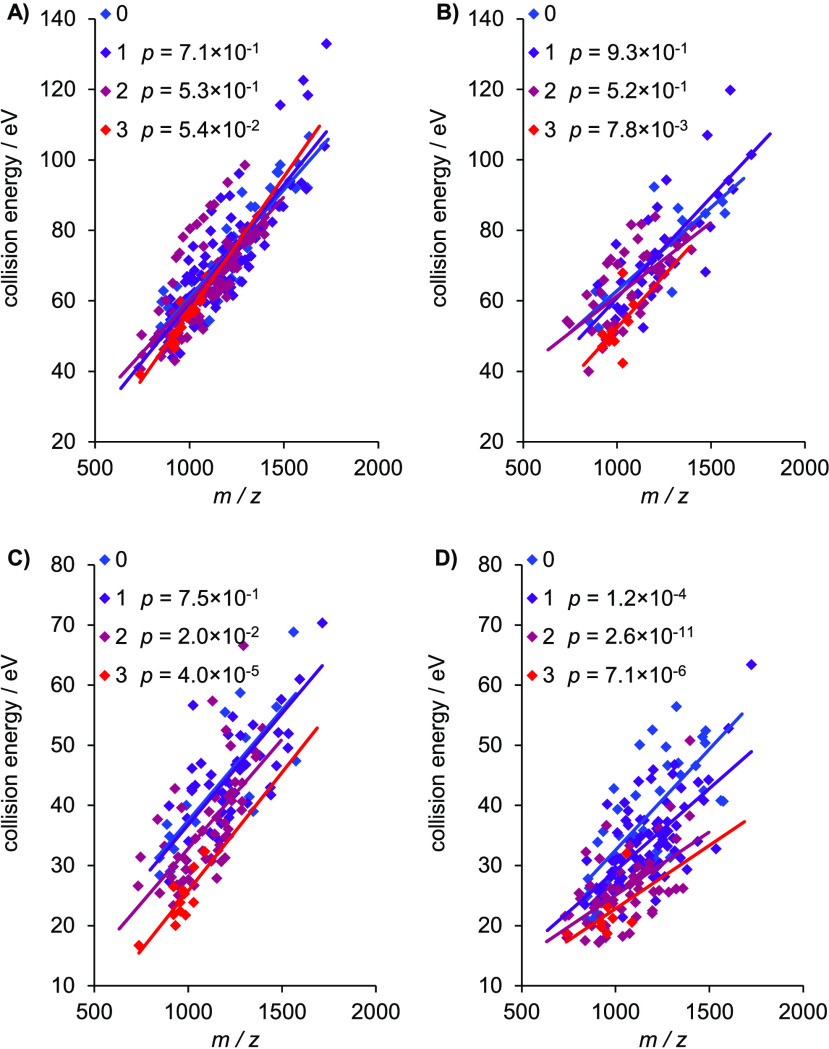
Optimal collision energies of *N*-glycopeptides in eV as a function of *m*/*z* for the various search engine scores and their dependence on the number of mobile protons. (A) Byonic, (B) pGlyco peptide, (C) pGlyco glycan, and (D) GlycoQuest. The *p* values show statistical significance compared to the case of zero mobile protons.

The effect of the number of mobile protons was studied similarly to that of hydrophobicity; here, the variable of interest is a categorical one. The number of mobile protons was expressed as the charge minus the number of all basic sites involving Lys, Arg, His, and N-terminus of *N*-glycopeptides.^59^ The search engine scores in the order of increasing dependence on the number of mobile protons are the following: Byonic and pGlyco peptide show no/slight differences, pGlyco glycan score is affected much more, and (as it can be seen in Figure 4) GlycoQuest optimum provides the largest dependence. Larger numbers of mobile protons require smaller optimal CE (see Figure 4). The explanation could be the following: mobile protons promote fragmentation of the intact glycopeptide resulting in glycan fragments, while peptide sequencing ions are coming from consecutive fragmentation processes from typically 1+ charged Y ions having no mobile protons anyway.^33^ Therefore, our findings on the effect of number of mobile protons corroborate the literature tested on only a few *N*-glycopeptide species.^33,46^ Further, the similarity between the behaviors of Byonic and pGlyco peptide scores reflects the peptide-centric nature of Byonic and the low dependence of Byonic score on glycan fragmentation.

The protons are also the charge carriers; therefore, we explored whether charge is a good indicator of the number of mobile protons of *N*-glycopeptides. Charge is known from MS without identification, which might be used in optimal CE setting choice. Our investigation revealed charge dependence of optimal CE for all scores but to a significantly different extent. The results are similar to the case of the number of mobile protons although the trends are less clear. The search engines in the order of increasing influence are the following: Byonic and pGlyco peptide scores behave similarly and show slight dependence. Charge has medium effect on optimal CE of pGlyco total and GlycoQuest scores. Finally, the pGlyco glycan score is affected the most by the charge states (see Figure S6).

We also showed that the charge and the number of mobile protons are well-correlated characteristics having a Pearson correlation of ca. 0.7 between them in the case of all investigated scores (about the strong correlation between charge and mobile protons see Figure S7). This strong correlation means that charge dependent CE setting choice can take into account the number of mobile protons and may be beneficial for confident oligosaccharide identification (peptide sequence determination seems independent). This should be set deliberately on QTof instruments while on Orbitrap mass spectrometers some charge dependence is already embedded in the NCE% definition.

#### Number of Sialic Acid Units

**Figure 5:**
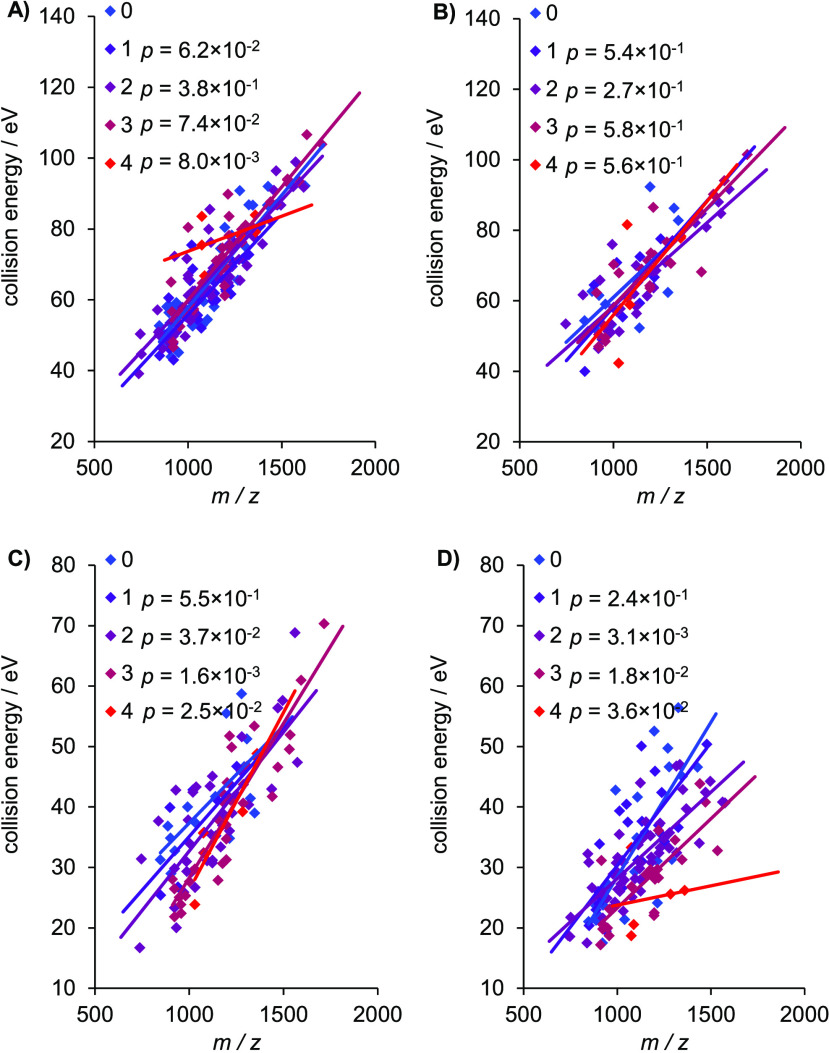
Optimal collision energies in eV as a function of *m*/*z* for the various search engine scores and their dependence on the number of sialic acid (SA) units. Only species without fucose are shown. (A) Byonic, (B) pGlyco peptide, (C) pGlyco glycan, and (D) GlycoQuest. The *p* values show statistical significance compared to the case of zero SA units.

The last characteristic that was found to affect optimal CE of *N*-glycopeptides in some cases is the number of sialic acid (SA) units of the oligosaccharide attached. The analysis of all species showed large residuals and outlier data points therefore we included only *N*-glycopeptides without the labile fucose unit to unravel the effect of number of SA units and the results involving all species are presented in Figure S8. In the order of increasing effect: The peptide score of pGlyco software is obviously not influenced by the number of SA units, while the Byonic score shows a slight dependence. In contrast, the scores describing deliberately glycan fragmentation (pGlyco glycan and GlycoQuest) both show systematic lowering of the optimal CE with increasing number of SA units (see Figure 5). The reason might be that SA is a labile group, i.e., dissociates easily,^6^ therefore for identifying glycans it is thus better to have less energy. Consequently, the confident identification of the number of SA units could be achieved at a somewhat lowered CE, which may be crucial because distorted sialylation is correlated with various diseases.

While the number of sialic acids cannot be determined before identifying the glycopeptide, the relatively large mass of the sialic acid units suggests that the dependence on this parameter may actually be related to the dependence on the *mass* of the *glycan part*. Our systematic tests indeed confirm the role of glycan mass (see below), which again provides a way to potentially take into account this parameter in an on-the-fly method to choose an optimal CE.

We also tested several further properties, namely, isoelectric point of peptide backbone, number of high mannose units in high mannose structures, number of antennae on complex structures, number of fucose units—neither of these showed a convincing trend, but in some cases, influence could be identified. Further, we checked the difference between glycopeptides with high-mannose and with complex glycans, but the two subgroups behaved the same way.

To conclude this section, we found various properties having a marked influence on the optimum energy in certain cases. The applied search engines display a range of behavior, and all trends can be understood if we consider that search engines display a range in terms of what they focus on: from the glycan to the peptide part. This is the first study to demonstrate a link between how differently focused search engines behave and the influences on the molecular level processes of fragmentation. Furthermore, we formulated a suggestion on how to consider the structural dependencies in practical CE setting choice.

### Effect of Sample Complexity

In the case of complex samples, mass spectrometry can have difficulties and limitations due to matrix effects (ME), which can negatively affect, among other things, reproducibility, accuracy or even sensitivity.^60^ When sensitivity is a key priority point (and this is the case for small amounts of difficult-to-ionize glycopeptides with a large diversity of different glycoforms), attempts should be made to minimize ME through MS parameters, chromatographic conditions, or optimized sample cleanup methods.

Although the glycopeptide-optimized enrichment step was a prominent step of sample preparation during this work, the complexity of HeLa and especially plasma samples was still crucial, which has a significant impact on MS measurements. Not only in terms of ionization efficiency and detectability but it also seems to affect the observed optimal CE values. Comparing the optimal CE values of the standard (AGP, fetuin and transferrin) and complex samples (HeLa and plasma together) (see Figure S9), an overall shift can be observed in the GlycoQuest search engine scores between the trend lines of the standard and complex samples. At 800 *m*/*z*, the shift seems to be approximately 5 eV, while at 1600 *m*/*z* this shift may be around 10 eV based on these data.

Examining the phenomenon through specific glycoform groups (see Figure S10), namely, singly and doubly sialylated glycoforms, a shift can be observed in the same direction and of the same magnitude between the optimal CE values of standard and complex samples using the GlycoQuest engine. Importantly, the trend between the optimal CE energy and the number of sialic acids remains consistent with the results generated on the whole data set (Figure 5/D): the optimal CE energy decreases with the increase of the number of SA units.

The evaluation of these measurements using the GlycoQuest search engine thus suggests that the optimal CE setting for *N*-glycopeptides is shifted toward higher energy for the complex sample. A notable shift also appears in the pGlyco glycan data, but the phenomenon is less prominent. For the more peptide-based scores (Byonic and pGlyco peptides), these data do not suggest a well-defined shift between the optimal CE data for the standard and complex samples. Thus, these results suggest that the complexity of the sample may primarily affect the fragmentation properties of the glycan moieties.

However, the standard glycoprotein mixture sample and the complex samples (HeLa and plasma) can generally be considered as two extreme cases among the glycoprotein samples for bioanalytical or even pharmaceutical applications. Thus, during practical use, the optimal collision energy settings for the *N*-glycopeptides of the examined proteolytically digested glycoprotein sample can be expected to fall within the energy range determined according to the presented optimization method.

### Systematic Testing of the Impact of Further Parameters

Besides studying individual structural properties selected by human judgment, we wanted to analyze if there are any further characteristics of the glycopeptide structure that have a significant influence on the optimum collision energy. To do so, we collected a large set of glycopeptide properties, including detailed amino acid and glycan composition, and used a machine learning method, lasso regression, to unbiasedly select the relevant variables.

We found that charge, *m*/*z* values, and masses of the peptide and glycan parts are the most important explanatory variables. Hydrophobicity and the position of the glycan-bearing amino acid in the sequence also contribute to a better fit. No further variables could be identified to bring about a significant advantage in explaining the optimum energy, in particular, no variable related to the exact composition (Table S6).

These observations are fully in line with those in the above section regarding peptide sequence and charge dependence. It appears that the influence of sialic acids may be interpreted as acting through the relative masses of the glycan/peptide parts; as indicated above, these might actually be more useful parameters in terms of workflow optimization.

### Can We Use This Knowledge to Improve Workflows?

As we already pointed out, the goal of a workflow is typically to *identify* glycopeptides in the sample, so from this practical perspective, the selection of a good collision energy cannot be based on the *exact knowledge* of the glycopeptide in question. Still, there may be properties that are much easier to determine than the full structure.

The *m*/*z* of the species to be identified is available from the MS1 spectrum. The charge *z* is also routinely available from the isotopic distribution in MS1. However, we envisioned that glycan mass and hydrophobicity could also be determined online.

It was shown that 80% of *N*-glycopeptide spectra taken under usual conditions contain characteristic Y0/Y1/Y2 fragment ions (full peptide + 0, 1, or 2 HexNAc units).^23^ Based on a preliminary MS/MS, and its online processing, the glycan and peptide masses can be calculated. We can also estimate the hydrophobicity of the species since it is well correlated with the retention time, which is known at the time of measurement. We can then use all this information and our multivariate regression results to choose a very precise collision energy for a subsequent detailed MS/MS.

This type of workflow would require adequate software support from the instrument manufacturers, but we thought our energy-dependent data set might help in assessing the potential gain it could provide. Using this data set, for each *N*-glycopeptide in our study, we estimated and compared (1) the score with which we could identify it using an optimal CE based on just the *m*/*z*, and (2) the score with which we could identify it using an optimal CE based on a regression against *m*/*z*, total mass, glycan mass, peptide-only *m*/*z*, and hydrophobicity. For each peptide, we took the score value from our actual measurement with the CE closest to the desired one.

Our data suggest that there might indeed be some potential gain from this approach, particularly for the more peptide-focused scores. Byonic score increases around 5% on average over all studied *N*-glycopeptides, while pGlyco score increases by 23% on average. Importantly, the gain is more significant for the *N*-glycopeptides originally having low score: for the bottom 1/5th of species in terms of score, the increase is around 15% for Byonic score and as much as around 100% for pGlyco peptide score. The gain is modest in the case of more glycan-focused scores, with pGlyco glycan score and GlycoQuest both having typically single-digit average percentage increases, even if we focus on the originally worst species.

On the other hand, our estimations are rather noisy due to the nature of the measured species-level score-CE relationships (cf. Figure S4), with individual *N*-glycopeptides displaying a very wide range of score gains and sometimes even losses. The average behavior still suggests that CE optimized beyond the search-engine and instrument-specific *m*/*z* dependence has some potential to improve identification confidence, particularly for species falling far from the *m*/*z* dependent trendline, but a more granular assessment is required in the future to corroborate the practical applicability.

## Conclusions

Robust mass spectrometric characterization of *N*-glycopeptides and the confidence of their identification depend on both experimental parameters (key: CE choice) and data evaluation procedure (key: the applied search engine). Our aim was to unravel structural properties influencing the optimal CE, to highlight differences between various search engines and to establish an advanced workflow incorporating the relevant characteristics in an online setup. CE dependent nano-LC-MS/MS experiments were carried out for several hundreds of *N*-glycopeptides covering various peptide sequences and glycan structures from glycoprotein standards and complex biological samples. Multiple search engine scores were investigated by using statistical methods and a machine learning approach. The following conclusions can be drawn from our findings:

- Trends in optimal CE, as well as trends in its dependence on various structural properties, highlight a range of behavior of different search engine scores. Byonic is confirmed to be peptide-focused, behaving similarly to the peptide score of pGlyco, as opposed to the glycan-focused cases: pGlyco’s glycan score and GlycoQuest score.
- Byonic’s lesser sensitivity to the glycan part and its known high-coverage nature might make it a useful tool for initial analysis of a sample, with the goal of creating a focused database where all potentially relevant species are included. In contrast, the more glycan-focused search engines might be used to obtain glycan structures with higher confidence.
- General linear models and lasso regression gave similar results in terms of what structural characteristics matter beyond *m*/*z*. Both approaches revealed that hydrophobicity of the peptide backbone and total charge (number of mobile protons) are important. The number of large SA units influences the optimal CE according to statistical analysis, which seems to correlate with the appearance of glycan and peptide masses separately in lasso regression.
- Based on the results of structural characteristics, we proposed a smart CE selection. In contrast to the classical choice, based on only *m*/*z* and charge values, our suggestion involves looking at the retention time to take hydrophobicity into account, and at the Y0/Y1/Y2 ion series of a preceding “scout MS2” for glycan mass estimation.
- Our data suggest that the smart CE approach would deliver significant gain for peptide-focused scores. The effect is especially significant in the case of originally low scored (just above the acceptance limit) *N*-glycopeptide species—the increase is around 15% for the Byonic score and as much as around 100% for the pGlyco peptide score. This may improve reproducibility between experiments of different laboratories and help in performing quantitative analysis due to lowering the number of missing values.

To the best of our knowledge, this study is the first to investigate influencing factors of optimal collision energies in this detail and on this large set of species at the same time. Beyond the insight from the obtained structure and search-engine dependence, we highlight a way to better experimental parameter selection, which appears essential in this research area to boost confidence and reproducibility.

## Supporting Information Available

Chemicals and details of enzymatic digestion; glycopeptide enrichment using acetone precipitation; first inclusion list of glycopeptides for the mixture of the 3 glycoprotein standards; second inclusion list of glycopeptides for the mixture of the 3 glycoprotein standards; inclusion list of glycopeptides for hela samples; inclusion list of glycopeptides for blood plasma samples; number of *n*-glycopeptide species as a function of peptide sequence; number of *N*-glycopeptide species as a function of glycan structure; *N*-glycopeptide identification using various search engines; determination of optimal CE setting using Serac; details of statistical methods;variables used in lasso regression; list of *N*-glycopeptide structures corresponding to the overlap of search engine identifications; score vs CE curves for QDQCIYNTTYLNVQR-HexNAc(5)Hex(6)NeuAc(2)4+; *N*-glycopeptide retention time as a function of peptide backbone hydrophobicity; optimal collision energies of *N*-glycopeptides in eV as a Function of *m*/*z*; correlation between the charge state and the average number of mobile protons; optimal collision energies of *N*-glycopeptides in eV as a function of *m*/*z*; optimal collision energies of *N*-glycopeptides for standards and complex samples; optimal collision energies of *N*-glycopeptides for GlycoQuest search engine for standards and complex samples with 1 or 2 sialic acid units; R2 and RMSE in regressions with various regularization parameter values (PDF)
