# Supplementary material for: Can We Boost N-Glycopeptide Identification Confidence? Smart Collision Energy Choice Taking into Account Structure and Search Engine
Source: J Am Soc Mass Spectrom. 2024 Jan 29;35(2):333–43. doi: 10.1021/jasms.3c00375 (PMC10853973; doi:10.1021/jasms.3c00375)
Supplement: Supplementary file 1 — js3c00375_si_001.pdf [file js3c00375_si_001.pdf]

Can we boost *N*-glycopeptide identification confidence? Smart collision energy choice taking into account structure and search engine

Helga Hevér,[1)] Andrea Xue,[1)] Kinga Nagy, [1,2)] Kinga Komka,[3)] Károly Vékey,[1)] László Drahos,[1)] Ágnes Révész[*1)]

[1)] MS Proteomics Research Group, HUN-REN Research Centre for Natural Sciences, Magyar Tudósok körútja 2., H-1117, Budapest, Hungary

[2)] Hevesy György PhD School of Chemistry, Eötvös Loránd University, Faculty of Science, Institute of Chemistry, Pázmány Péter sétány 1/A, Budapest, H-1117, Hungary

[3)] Department of Chemical and Environmental Process Engineering, Budapest University of Technology and Economics, Budapest, H-1111, Hungary

*E-mail: revesz.agnes@ttk.hu

Supporting Information

Table of Contents

Further Experimental Details

- **Material S1: Chemicals and Details of Enzymatic Digestion**
- **Material S2: Glycopeptide Enrichment Using Acetone Precipitation**
- **Table S1: First Inclusion List of Glycopeptides for the Mixture of the 3 Glycoprotein Standards**
- **Table S2: Second Inclusion List of Glycopeptides for the Mixture of the 3 Glycoprotein Standards**
- **Table S3: The Inclusion List of Glycopeptides for HeLa Samples**
- **Table S4: The Inclusion List of Glycopeptides for Blood Plasma Samples**
- **Figure S1: The Number of *N*-glycopeptide Species as a Function of Peptide Sequence**
- **Figure S2: The Number of *N*-glycopeptide Species as a Function of Glycan Structure**
- **Material S3: *N*-glycopeptide Identification Using Various Search Engines**
- **Material S4: Determination of Optimal CE setting Using Serac**
- **Table S5: Details of Statistical Methods**
- **Material S5: Variables Used in Lasso Regression**

***N*-glycopeptide Level Optimal Collision Energy**

- **Figure S3: List of *N*-glycopeptide Structures Corresponding to the Overlap of Search Engine Identifications**
- **Figure S4: Score vs. CE Curves for QDQCIYNTTYLNVQR-HexNAc(5)Hex(6)NeuAc(2)$^{4+}$**

Correlation between Retention Time and Peptide Backbone Hydrophobicity

- **Figure S5: *N*-glycopeptide Retention Time as a Function of Peptide Backbone Hydrophobicity**

Charge Dependence of the Optimal CE for the Various Search Engine Scores

- **Figure S6: Optimal Collision Energies of *N*-glycopeptides in eV as a Function of *m/z***
- **Figure S7: Correlation between the Charge State and the Average Number of Mobile Protons**

Effect of Number of SA Units on the Optimal CE for the Various Search Engine Scores

- **Figure S8: Optimal Collision Energies of *N*-glycopeptides in eV as a Function of *m/z***

Effect of Sample Complexity

Figure S9: Optimal Collision Energies of *N*-glycopeptides for Standards and Complex Samples

Figure S10: Optimal Collision Energies of *N*-glycopeptides for GlycoQuest Search Engine for Standards and Complex Samples with 1 or 2 Sialic Acid Units

Results from Lasso Regression

Table S6: R^2 and RMSE In Regressions with Various Regularization Parameter Values

Further Experimental Details

Material S1: Chemicals and Details of Enzymatic Digestion

Chemical Reagents

Unless otherwise stated, reagents and consumables were from Sigma-Aldrich (Sigma-Aldrich Kft., Budapest, Hungary). RapiGest SF was purchased from Waters (Milford, MA), trypsin/Lys-C mix and trypsin digestion enzymes were from Promega (Madison, WI). α-1 acid glycoprotein (AGP), fetuin and transferrin glycoprotein standards were obtained from Sigma-Aldrich (Sigma-Aldrich Kft., Budapest, Hungary), HeLa tryptic digest standard was from Thermo Fisher Scientific (Waltham, MA).

Enzymatic Digestion of Glycoprotein Standards

We worked with glycoprotein aliquots of 1 nmol. Denaturation of the samples was performed by Rapigest SF, the S–S bridges were reduced by dithiothreitol followed by alkylation using iodoacetamide in dark. Then the samples were digested first by Lys-C/trypsin mixture (1 h) followed by digestion using trypsin (3 h). The appropriate pH was set using ammonium bicarbonate buffer solution. Digestion was quenched by the addition of formic acid. The digests of glycoprotein standards were divided into 5 portions (i.e., aliquots of 200 pmol) and were dried in SpeedVac. From each sample, 1 aliquot was dissolved in injection solvent (98% water, 2% acetonitrile and 0.1% formic acid) prior to nano-LC-MS/MS analysis.

Material S2: Glycopeptide Enrichment Using Acetone Precipitation

The samples were dissolved in 15 µL water + 1% formic acid, then 150 µL ice-cold acetone was added. The solution was stored at −20 °C overnight resulting in the formation of a pellet which may contribute to increasing the ratio of glycopeptides. The sample was centrifuged at 12 000 g for 10 minutes. The supernatant containing most of the peptides was removed by pipetting. The pellet fraction was dried in SpeedVac and re-dissolved in solvent (98% water, 2% acetonitrile and 0.1% formic acid) prior to nano-LC-MS/MS analysis.

Table S1. First inclusion list of glycopeptides for the mixture of the 3 glycoprotein standards according to *m*/*z*, peptide sequence, glycan composition, charge, protein ID and accession number.

m*/*z	**Peptide sequence**	**Glycan composition**	**Charge**	**Accession number**	**Protein ID**
1219.7872	NEEYNK	HexNAc(5)Hex(6)NeuAc(3)	3	P02763	A1AG1_HUMAN
946.6175	ENGTISR	HexNAc(5)Hex(6)Fuc(1)NeuAc(3)	4	P02763	A1AG1_HUMAN
910.1030	ENGTISR	HexNAc(5)Hex(6)NeuAc(3)	4	P02763	A1AG1_HUMAN
1261.8209	ENGTISR	HexNAc(5)Hex(6)Fuc(1)NeuAc(3)	3	P02763	A1AG1_HUMAN
1122.7555	NEEYNK	HexNAc(5)Hex(6)NeuAc(2)	3	P02763	A1AG1_HUMAN
1213.1349	ENGTISR	HexNAc(5)Hex(6)NeuAc(3)	3	P02763	A1AG1_HUMAN
1164.7889	ENGTISR	HexNAc(5)Hex(6)Fuc(1)NeuAc(2)	3	P02763	A1AG1_HUMAN
1116.1029	ENGTISR	HexNAc(5)Hex(6)NeuAc(2)	3	P02763	A1AG1_HUMAN
873.8435	ENGTISR	HexNAc(5)Hex(6)Fuc(1)NeuAc(2)	4	P02763	A1AG1_HUMAN
994.3922	ENGTISR	HexNAc(4)Hex(5)NeuAc(2)	3	P02763	A1AG1_HUMAN
1025.7235	NEEYNK	HexNAc(5)Hex(6)NeuAc(1)	3	P02763	A1AG1_HUMAN
1019.0712	ENGTISR	HexNAc(5)Hex(6)NeuAc(1)	3	P02763	A1AG1_HUMAN
897.3605	ENGTISR	HexNAc(4)Hex(5)NeuAc(1)	3	P02763	A1AG1_HUMAN
1231.9980	QDQCIYNTTYLNVQR	HexNAc(7)Hex(8)NeuAc(1)	4	P02763	A1AG1_HUMAN
1141.9572	QNQCFYNSSYLNVQR	HexNAc(6)Hex(7)NeuAc(1)	4	P19652	A1AG2_HUMAN
1050.6742	QNQCFYNSSYLNVQR	HexNAc(5)Hex(6)NeuAc(1)	4	P19652	A1AG2_HUMAN
1177.2295	QDQCIYNTTYLNVQR	HexNAc(6)Hex(7)Fuc(1)NeuAc(1)	4	P02763	A1AG1_HUMAN
912.7734	QDQCIYNTTYLNVQR	HexNAc(6)Hex(7)NeuAc(1)	5	P02763	A1AG1_HUMAN
1140.7150	QDQCIYNTTYLNVQR	HexNAc(6)Hex(7)NeuAc(1)	4	P02763	A1AG1_HUMAN

1049.4320	QDQCIYNTTYLNVQR	HexNAc(5)Hex(6)NeuAc(1)	4	P02763	A1AG1_HUMAN
1214.7312	QNQCFYNSSYLNVQR	HexNAc(6)Hex(7)NeuAc(2)	4	P19652	A1AG2_HUMAN
1159.9625	QNQCFYNSSYLNVQR	HexNAc(5)Hex(6)Fuc(1)NeuAc(2)	4	P19652	A1AG2_HUMAN
1123.4480	QNQCFYNSSYLNVQR	HexNAc(5)Hex(6)NeuAc(2)	4	P19652	A1AG2_HUMAN
1251.2457	QNQCFYNSSYLNVQR	HexNAc(6)Hex(7)Fuc(1)NeuAc(2)	4	P19652	A1AG2_HUMAN
1250.0032	QDQCIYNTTYLNVQR	HexNAc(6)Hex(7)Fuc(1)NeuAc(2)	4	P02763	A1AG1_HUMAN
1000.2040	QDQCIYNTTYLNVQR	HexNAc(6)Hex(7)Fuc(1)NeuAc(2)	5	P02763	A1AG1_HUMAN
1213.4890	QDQCIYNTTYLNVQR	HexNAc(6)Hex(7)NeuAc(2)	4	P02763	A1AG1_HUMAN
970.9926	QDQCIYNTTYLNVQR	HexNAc(6)Hex(7)NeuAc(2)	5	P02763	A1AG1_HUMAN
1617.6495	QDQCIYNTTYLNVQR	HexNAc(6)Hex(7)NeuAc(2)	3	P02763	A1AG1_HUMAN
1158.7202	QDQCIYNTTYLNVQR	HexNAc(5)Hex(6)Fuc(1)NeuAc(2)	4	P02763	A1AG1_HUMAN
1122.2057	QDQCIYNTTYLNVQR	HexNAc(5)Hex(6)NeuAc(2)	4	P02763	A1AG1_HUMAN
897.9660	QDQCIYNTTYLNVQR	HexNAc(5)Hex(6)NeuAc(2)	5	P02763	A1AG1_HUMAN
1495.9385	QDQCIYNTTYLNVQR	HexNAc(5)Hex(6)NeuAc(2)	3	P02763	A1AG1_HUMAN
1030.9227	QDQCIYNTTYLNVQR	HexNAc(4)Hex(5)NeuAc(2)	4	P02763	A1AG1_HUMAN
939.6457	CGLVPVLAENYNK	HexNAc(5)Hex(6)NeuAc(1)	4	P02787	TRFE_HUMAN
1252.5252	CGLVPVLAENYNK	HexNAc(5)Hex(6)NeuAc(1)	3	P02787	TRFE_HUMAN
1324.0195	QNQCFYNSSYLNVQR	HexNAc(6)Hex(7)Fuc(1)NeuAc(3)	4	P19652	A1AG2_HUMAN
1130.8145	CGLVPVLAENYNK	HexNAc(4)Hex(5)NeuAc(1)	3	P02787	TRFE_HUMAN
848.3627	CGLVPVLAENYNK	HexNAc(4)Hex(5)NeuAc(1)	4	P02787	TRFE_HUMAN
1232.7365	QNQCFYNSSYLNVQR	HexNAc(5)Hex(6)Fuc(1)NeuAc(3)	4	P19652	A1AG2_HUMAN
957.1790	QNQCFYNSSYLNVQR	HexNAc(5)Hex(6)NeuAc(3)	5	P19652	A1AG2_HUMAN
1287.5050	QNQCFYNSSYLNVQR	HexNAc(6)Hex(7)NeuAc(3)	4	P19652	A1AG2_HUMAN
1030.2054	QNQCFYNSSYLNVQR	HexNAc(6)Hex(7)NeuAc(3)	5	P19652	A1AG2_HUMAN
1196.2220	QNQCFYNSSYLNVQR	HexNAc(5)Hex(6)NeuAc(3)	4	P19652	A1AG2_HUMAN
1059.4170	QNQCFYNSSYLNVQR	HexNAc(6)Hex(7)Fuc(1)NeuAc(3)	5	P19652	A1AG2_HUMAN
1029.2116	QDQCIYNTTYLNVQR	HexNAc(6)Hex(7)NeuAc(3)	5	P02763	A1AG1_HUMAN
1322.7772	QDQCIYNTTYLNVQR	HexNAc(6)Hex(7)Fuc(1)NeuAc(3)	4	P02763	A1AG1_HUMAN
1286.2627	QDQCIYNTTYLNVQR	HexNAc(6)Hex(7)NeuAc(3)	4	P02763	A1AG1_HUMAN
1194.9797	QDQCIYNTTYLNVQR	HexNAc(5)Hex(6)NeuAc(3)	4	P02763	A1AG1_HUMAN
1231.4942	QDQCIYNTTYLNVQR	HexNAc(5)Hex(6)Fuc(1)NeuAc(3)	4	P02763	A1AG1_HUMAN
985.3968	QDQCIYNTTYLNVQR	HexNAc(5)Hex(6)Fuc(1)NeuAc(3)	5	P02763	A1AG1_HUMAN
1714.6812	QDQCIYNTTYLNVQR	HexNAc(6)Hex(7)NeuAc(3)	3	P02763	A1AG1_HUMAN
1592.9705	QDQCIYNTTYLNVQR	HexNAc(5)Hex(6)NeuAc(3)	3	P02763	A1AG1_HUMAN
956.1852	QDQCIYNTTYLNVQR	HexNAc(5)Hex(6)NeuAc(3)	5	P02763	A1AG1_HUMAN
1058.4232	QDQCIYNTTYLNVQR	HexNAc(6)Hex(7)Fuc(1)NeuAc(3)	5	P02763	A1AG1_HUMAN
1349.5569	CGLVPVLAENYNK	HexNAc(5)Hex(6)NeuAc(2)	3	P02787	TRFE_HUMAN
1012.4195	CGLVPVLAENYNK	HexNAc(5)Hex(6)NeuAc(2)	4	P02787	TRFE_HUMAN
1110.4655	KLCPDCPLLAPLNDSR	HexNAc(5)Hex(6)NeuAc(2)	4	P12763	FETUA_BOVIN
1227.8462	CGLVPVLAENYNK	HexNAc(4)Hex(5)NeuAc(2)	3	P02787	TRFE_HUMAN
921.1365	CGLVPVLAENYNK	HexNAc(4)Hex(5)NeuAc(2)	4	P02787	TRFE_HUMAN
737.1106	CGLVPVLAENYNK	HexNAc(4)Hex(5)NeuAc(2)	5	P02787	TRFE_HUMAN
1841.2656	CGLVPVLAENYNK	HexNAc(4)Hex(5)NeuAc(2)	2	P02787	TRFE_HUMAN
1218.8442	LCPDCPLLAPLNDSR	HexNAc(4)Hex(5)NeuAc(1)	3	P12763	FETUA_BOVIN

1144.4702	QQQHLFGSNVTDCSGNFCLFR	HexNAc(4)Hex(5)Fuc(1)NeuAc(1)	4	P02787	TRFE_HUMAN
915.7776	QQQHLFGSNVTDCSGNFCLFR	HexNAc(4)Hex(5)Fuc(1)NeuAc(1)	5	P02787	TRFE_HUMAN
1476.9385	QQQHLFGSNVTDCSGNFCLFR	HexNAc(4)Hex(5)NeuAc(1)	3	P02787	TRFE_HUMAN
1107.9557	QQQHLFGSNVTDCSGNFCLFR	HexNAc(4)Hex(5)NeuAc(1)	4	P02787	TRFE_HUMAN
886.5660	QQQHLFGSNVTDCSGNFCLFR	HexNAc(4)Hex(5)NeuAc(1)	5	P02787	TRFE_HUMAN
1486.8342	QDQCIYNTTYLNVQR	HexNAc(7)Hex(8)Fuc(1)NeuAc(4)	4	P02763	A1AG1_HUMAN
946.7930	KLCPDCPLLAPLNDSR	HexNAc(5)Hex(6)NeuAc(3)	5	P12763	FETUA_BOVIN
1088.4244	QNQCFYNSSYLNVQR	HexNAc(6)Hex(7)NeuAc(4)	5	P19652	A1AG2_HUMAN
1183.2395	KLCPDCPLLAPLNDSR	HexNAc(5)Hex(6)NeuAc(3)	4	P12763	FETUA_BOVIN
1360.2787	QNQCFYNSSYLNVQR	HexNAc(6)Hex(7)NeuAc(4)	4	P19652	A1AG2_HUMAN
1396.7932	QNQCFYNSSYLNVQR	HexNAc(6)Hex(7)Fuc(1)NeuAc(4)	4	P19652	A1AG2_HUMAN
1078.4417	LCPDCPLLAPLNDSR	HexNAc(5)Hex(6)NeuAc(2)	4	P12763	FETUA_BOVIN
1437.5865	LCPDCPLLAPLNDSR	HexNAc(5)Hex(6)NeuAc(2)	3	P12763	FETUA_BOVIN
987.1587	LCPDCPLLAPLNDSR	HexNAc(4)Hex(5)NeuAc(2)	4	P12763	FETUA_BOVIN
1395.5510	QDQCIYNTTYLNVQR	HexNAc(6)Hex(7)Fuc(1)NeuAc(4)	4	P02763	A1AG1_HUMAN
1359.0365	QDQCIYNTTYLNVQR	HexNAc(6)Hex(7)NeuAc(4)	4	P02763	A1AG1_HUMAN
1087.4306	QDQCIYNTTYLNVQR	HexNAc(6)Hex(7)NeuAc(4)	5	P02763	A1AG1_HUMAN
1315.8759	LCPDCPLLAPLNDSR	HexNAc(4)Hex(5)NeuAc(2)	3	P12763	FETUA_BOVIN
1308.5272	QQQHLFGSNVTDCSGNFCLFR	HexNAc(5)Hex(6)Fuc(1)NeuAc(2)	4	P02787	TRFE_HUMAN
1217.2442	QQQHLFGSNVTDCSGNFCLFR	HexNAc(4)Hex(5)Fuc(1)NeuAc(2)	4	P02787	TRFE_HUMAN
973.9968	QQQHLFGSNVTDCSGNFCLFR	HexNAc(4)Hex(5)Fuc(1)NeuAc(2)	5	P02787	TRFE_HUMAN
1622.6565	QQQHLFGSNVTDCSGNFCLFR	HexNAc(4)Hex(5)Fuc(1)NeuAc(2)	3	P02787	TRFE_HUMAN
1036.4282	WFYIASAFRNEEYNK	HexNAc(4)Hex(5)NeuAc(2)	4	P02763	A1AG1_HUMAN
1017.8116	QQQHLFGSNVTDCSGNFCLFR	HexNAc(5)Hex(6)NeuAc(2)	5	P02787	TRFE_HUMAN
1272.0127	QQQHLFGSNVTDCSGNFCLFR	HexNAc(5)Hex(6)NeuAc(2)	4	P02787	TRFE_HUMAN
1573.9705	QQQHLFGSNVTDCSGNFCLFR	HexNAc(4)Hex(5)NeuAc(2)	3	P02787	TRFE_HUMAN
944.7852	QQQHLFGSNVTDCSGNFCLFR	HexNAc(4)Hex(5)NeuAc(2)	5	P02787	TRFE_HUMAN
1180.7297	QQQHLFGSNVTDCSGNFCLFR	HexNAc(4)Hex(5)NeuAc(2)	4	P02787	TRFE_HUMAN
1236.9995	WFYIASAFRNEEYNK	HexNAc(5)Hex(6)Fuc(1)NeuAc(3)	4	P02763	A1AG1_HUMAN
1200.4850	WFYIASAFRNEEYNK	HexNAc(5)Hex(6)NeuAc(3)	4	P02763	A1AG1_HUMAN
1105.2422	QQQHLFGSNVTDCSGNFCLFR	HexNAc(5)Hex(6)Fuc(1)NeuAc(3)	5	P02787	TRFE_HUMAN
1381.3010	QQQHLFGSNVTDCSGNFCLFR	HexNAc(5)Hex(6)Fuc(1)NeuAc(3)	4	P02787	TRFE_HUMAN
1076.0306	QQQHLFGSNVTDCSGNFCLFR	HexNAc(5)Hex(6)NeuAc(3)	5	P02787	TRFE_HUMAN
1344.7865	QQQHLFGSNVTDCSGNFCLFR	HexNAc(5)Hex(6)NeuAc(3)	4	P02787	TRFE_HUMAN
1534.6185	LCPDCPLLAPLNDSR	HexNAc(5)Hex(6)NeuAc(3)	3	P12763	FETUA_BOVIN
921.1740	LCPDCPLLAPLNDSR	HexNAc(5)Hex(6)NeuAc(3)	5	P12763	FETUA_BOVIN
1151.2157	LCPDCPLLAPLNDSR	HexNAc(5)Hex(6)NeuAc(3)	4	P12763	FETUA_BOVIN
1249.1404	RPTGEVYDIEIDTLETTCHVLDPTPLANCSVR	HexNAc(5)Hex(6)NeuAc(2)	5	P12763	FETUA_BOVIN
1597.6882	RPTGEVYDIEIDTLETTCHVLDPTPLANCSVR	HexNAc(5)Hex(6)Fuc(1)NeuAc(2)	4	P12763	FETUA_BOVIN
1561.1737	RPTGEVYDIEIDTLETTCHVLDPTPLANCSVR	HexNAc(5)Hex(6)NeuAc(2)	4	P12763	FETUA_BOVIN
1469.8905	RPTGEVYDIEIDTLETTCHVLDPTPLANCSVR	HexNAc(4)Hex(5)NeuAc(2)	4	P12763	FETUA_BOVIN
1176.1138	RPTGEVYDIEIDTLETTCHVLDPTPLANCSVR	HexNAc(4)Hex(5)NeuAc(2)	5	P12763	FETUA_BOVIN
1633.9475	RPTGEVYDIEIDTLETTCHVLDPTPLANCSVR	HexNAc(5)Hex(6)NeuAc(3)	4	P12763	FETUA_BOVIN
1385.6050	SVQEIQATFFYFTPNKTEDTIFLR	HexNAc(6)Hex(7)NeuAc(1)	4	P02763	A1AG1_HUMAN

1307.3594	RPTGEVYDIEIDTLETTCHVLDPTPLANCSVR	HexNAc(5)Hex(6)NeuAc(3)	5	P12763	FETUA_BOVIN
1294.3220	SVQEIQATFFYFTPNKTEDTIFLR	HexNAc(5)Hex(6)NeuAc(1)	4	P02763	A1AG1_HUMAN
1203.0387	SVQEIQATFFYFTPNKTEDTIFLR	HexNAc(4)Hex(5)NeuAc(1)	4	P02763	A1AG1_HUMAN
1324.6012	VVHAVEVALATFNAESNGSYLQLVEISR	HexNAc(5)Hex(6)NeuAc(1)	4	P12763	FETUA_BOVIN
1458.3787	SVQEIQATFFYFTPNKTEDTIFLR	HexNAc(6)Hex(7)NeuAc(2)	4	P02763	A1AG1_HUMAN
1166.9044	SVQEIQATFFYFTPNKTEDTIFLR	HexNAc(6)Hex(7)NeuAc(2)	5	P02763	A1AG1_HUMAN
1123.0896	SVQEIQATFFYFTPNKTEDTIFLR	HexNAc(5)Hex(6)Fuc(1)NeuAc(2)	5	P02763	A1AG1_HUMAN
1093.8780	SVQEIQATFFYFTPNKTEDTIFLR	HexNAc(5)Hex(6)NeuAc(2)	5	P02763	A1AG1_HUMAN
1367.0957	SVQEIQATFFYFTPNKTEDTIFLR	HexNAc(5)Hex(6)NeuAc(2)	4	P02763	A1AG1_HUMAN
1020.8516	SVQEIQATFFYFTPNKTEDTIFLR	HexNAc(4)Hex(5)NeuAc(2)	5	P02763	A1AG1_HUMAN
1275.8127	SVQEIQATFFYFTPNKTEDTIFLR	HexNAc(4)Hex(5)NeuAc(2)	4	P02763	A1AG1_HUMAN
1118.1016	VVHAVEVALATFNAESNGSYLQLVEISR	HexNAc(5)Hex(6)NeuAc(2)	5	P12763	FETUA_BOVIN
1397.3752	VVHAVEVALATFNAESNGSYLQLVEISR	HexNAc(5)Hex(6)NeuAc(2)	4	P12763	FETUA_BOVIN
1225.1236	SVQEIQATFFYFTPNKTEDTIFLR	HexNAc(6)Hex(7)NeuAc(3)	5	P02763	A1AG1_HUMAN
1531.1527	SVQEIQATFFYFTPNKTEDTIFLR	HexNAc(6)Hex(7)NeuAc(3)	4	P02763	A1AG1_HUMAN
1476.3840	SVQEIQATFFYFTPNKTEDTIFLR	HexNAc(5)Hex(6)Fuc(1)NeuAc(3)	4	P02763	A1AG1_HUMAN
1439.8695	SVQEIQATFFYFTPNKTEDTIFLR	HexNAc(5)Hex(6)NeuAc(3)	4	P02763	A1AG1_HUMAN
1152.0970	SVQEIQATFFYFTPNKTEDTIFLR	HexNAc(5)Hex(6)NeuAc(3)	5	P02763	A1AG1_HUMAN
1470.1490	VVHAVEVALATFNAESNGSYLQLVEISR	HexNAc(5)Hex(6)NeuAc(3)	4	P12763	FETUA_BOVIN
1959.8629	VVHAVEVALATFNAESNGSYLQLVEISR	HexNAc(5)Hex(6)NeuAc(3)	3	P12763	FETUA_BOVIN
1205.5322	VVHAVEVALATFNAESNGSYLQLVEISR	HexNAc(5)Hex(6)Fuc(1)NeuAc(3)	5	P12763	FETUA_BOVIN
1176.3206	VVHAVEVALATFNAESNGSYLQLVEISR	HexNAc(5)Hex(6)NeuAc(3)	5	P12763	FETUA_BOVIN
980.4351	VVHAVEVALATFNAESNGSYLQLVEISR	HexNAc(5)Hex(6)NeuAc(3)	6	P12763	FETUA_BOVIN
1283.3426	SVQEIQATFFYFTPNKTEDTIFLR	HexNAc(6)Hex(7)NeuAc(4)	5	P02763	A1AG1_HUMAN
1634.2060	VVHAVEVALATFNAESNGSYLQLVEISR	HexNAc(6)Hex(7)NeuAc(4)	4	P12763	FETUA_BOVIN
1029.8188	QDQCIYNTTYLNVQR	HexNAc(8)Hex(9)Fuc(1)	5	P02763	A1AG1_HUMAN
1019.1825	KLCPDCPLLAPLNDSR	HexNAc(4)Hex(5)NeuAc(2)	4	P12763	FETUA_BOVIN
1134.8570	QQQHLFGSNVTDCSGNFCLFR	HexNAc(7)Hex(8)Fuc(1)NeuAc(1)	5	P02787	TRFE_HUMAN
1336.5710	RPTGEVYDIEIDTLETTCHVLDPTPLANCSVR	HexNAc(5)Hex(6)Fuc(1)NeuAc(3)	5	P12763	FETUA_BOVIN
1195.9940	SVQEIQATFFYFTPNK	HexNAc(5)Hex(6)NeuAc(3)	4	P02763	A1AG1_HUMAN
1181.3086	SVQEIQATFFYFTPNKTEDTIFLR	HexNAc(5)Hex(6)Fuc(1)NeuAc(3)	5	P02763	A1AG1_HUMAN
1312.5542	SVQEIQATFFYFTPNKTEDTIFLR	HexNAc(6)Hex(7)Fuc(1)NeuAc(4)	5	P02763	A1AG1_HUMAN
1045.0750	VVHAVEVALATFNAESNGSYLQLVEISR	HexNAc(4)Hex(5)NeuAc(2)	5	P12763	FETUA_BOVIN
931.9192	VVHAVEVALATFNAESNGSYLQLVEISR	HexNAc(5)Hex(6)NeuAc(2)	6	P12763	FETUA_BOVIN

Table S2. Second inclusion list of glycopeptides for the mixture of the 3 glycoprotein standards according to *m*/*z*, peptide sequence, glycan composition, charge, protein ID and accession number.

m*/*z	**Peptide sequence**	**Glycan composition**	**Charge**	**Accession number**	**Protein ID**
1723.9849	ENGTISR	HexNAc(8)Hex(9)Fuc(1)NeuAc(4)	3	P02763	A1AG1_HUMAN
1293.2405	ENGTISR	HexNAc(8)Hex(9)Fuc(1)NeuAc(4)	4	P02763	A1AG1_HUMAN
1201.9575	ENGTISR	HexNAc(7)Hex(8)Fuc(1)NeuAc(4)	4	P02763	A1AG1_HUMAN
1602.2742	ENGTISR	HexNAc(7)Hex(8)Fuc(1)NeuAc(4)	3	P02763	A1AG1_HUMAN

1480.5632	ENGTISR	HexNAc(6)Hex(7)Fuc(1)NeuAc(4)	3	P02763	A1AG1_HUMAN
1110.6742	ENGTISR	HexNAc(6)Hex(7)Fuc(1)NeuAc(4)	4	P02763	A1AG1_HUMAN
1074.1597	ENGTISR	HexNAc(6)Hex(7)NeuAc(4)	4	P02763	A1AG1_HUMAN
1626.9529	ENGTISR	HexNAc(8)Hex(9)Fuc(1)NeuAc(3)	3	P02763	A1AG1_HUMAN
1129.1835	ENGTISR	HexNAc(7)Hex(8)Fuc(1)NeuAc(3)	4	P02763	A1AG1_HUMAN
1001.3860	ENGTISR	HexNAc(6)Hex(7)NeuAc(3)	4	P02763	A1AG1_HUMAN
1037.9005	ENGTISR	HexNAc(6)Hex(7)Fuc(1)NeuAc(3)	4	P02763	A1AG1_HUMAN
965.1265	ENGTISR	HexNAc(6)Hex(7)Fuc(1)NeuAc(2)	4	P02763	A1AG1_HUMAN
928.6122	ENGTISR	HexNAc(6)Hex(7)NeuAc(2)	4	P02763	A1AG1_HUMAN
837.3290	ENGTISR	HexNAc(5)Hex(6)NeuAc(2)	4	P02763	A1AG1_HUMAN
746.0460	ENGTISR	HexNAc(4)Hex(5)NeuAc(2)	4	P02763	A1AG1_HUMAN
1189.4679	ENGTISR	HexNAc(6)Hex(7)Fuc(1)NeuAc(1)	3	P02763	A1AG1_HUMAN
1140.7819	ENGTISR	HexNAc(6)Hex(7)NeuAc(1)	3	P02763	A1AG1_HUMAN
855.8382	ENGTISR	HexNAc(6)Hex(7)NeuAc(1)	4	P02763	A1AG1_HUMAN
1067.7572	ENGTISR	HexNAc(5)Hex(6)Fuc(1)NeuAc(1)	3	P02763	A1AG1_HUMAN
1231.9980	QDQCIYNTTYLNVQR	HexNAc(7)Hex(8)NeuAc(1)	4	P02763	A1AG1_HUMAN
1050.6742	QNQCFYNSSYLNVQR	HexNAc(5)Hex(6)NeuAc(1)	4	P19652	A1AG2_HUMAN
912.7734	QDQCIYNTTYLNVQR	HexNAc(6)Hex(7)NeuAc(1)	5	P02763	A1AG1_HUMAN
1000.2040	QDQCIYNTTYLNVQR	HexNAc(6)Hex(7)Fuc(1)NeuAc(2)	5	P02763	A1AG1_HUMAN
1179.5005	CGLVPVLAENYNK	HexNAc(4)Hex(5)Fuc(1)NeuAc(1)	3	P02787	TRFE_HUMAN
884.8772	CGLVPVLAENYNK	HexNAc(4)Hex(5)Fuc(1)NeuAc(1)	4	P02787	TRFE_HUMAN
1059.4170	QNQCFYNSSYLNVQR	HexNAc(6)Hex(7)Fuc(1)NeuAc(3)	5	P19652	A1AG2_HUMAN
1841.2656	CGLVPVLAENYNK	HexNAc(4)Hex(5)NeuAc(2)	2	P02787	TRFE_HUMAN
1276.5322	CGLVPVLAENYNK	HexNAc(4)Hex(5)Fuc(1)NeuAc(2)	3	P02787	TRFE_HUMAN
957.6510	CGLVPVLAENYNK	HexNAc(4)Hex(5)Fuc(1)NeuAc(2)	4	P02787	TRFE_HUMAN
1486.8342	QDQCIYNTTYLNVQR	HexNAc(7)Hex(8)Fuc(1)NeuAc(4)	4	P02763	A1AG1_HUMAN
1183.9985	KLCPDCPLLAPLNDSR	HexNAc(7)Hex(8)Fuc(1)	4	P12763	FETUA_BOVIN
1396.7932	QNQCFYNSSYLNVQR	HexNAc(6)Hex(7)Fuc(1)NeuAc(4)	4	P19652	A1AG2_HUMAN
1016.6727	QQQHLFGSNVTDCSGNFCLFR	HexNAc(3)Hex(4)NeuAc(1)	4	P02787	TRFE_HUMAN
1236.9995	WFYIASAFRNEEYNK	HexNAc(5)Hex(6)Fuc(1)NeuAc(3)	4	P02763	A1AG1_HUMAN
921.1740	LCPDCPLLAPLNDSR	HexNAc(5)Hex(6)NeuAc(3)	5	P12763	FETUA_BOVIN
1597.6882	RPTGEVYDIEIDTLETTCHVLDPTPLANCSV<br>R	HexNAc(5)Hex(6)Fuc(1)NeuAc(2)	4	P12763	FETUA_BOVIN
1385.6050	SVQEIQATFFYFTPNKTEDTIFLR	HexNAc(6)Hex(7)NeuAc(1)	4	P02763	A1AG1_HUMAN
1294.3220	SVQEIQATFFYFTPNKTEDTIFLR	HexNAc(5)Hex(6)NeuAc(1)	4	P02763	A1AG1_HUMAN
1531.1527	SVQEIQATFFYFTPNKTEDTIFLR	HexNAc(6)Hex(7)NeuAc(3)	4	P02763	A1AG1_HUMAN
1959.8629	VVHAVEVALATFNAESNGSYLQLVEISR	HexNAc(5)Hex(6)NeuAc(3)	3	P12763	FETUA_BOVIN
1205.5322	VVHAVEVALATFNAESNGSYLQLVEISR	HexNAc(5)Hex(6)Fuc(1)NeuAc(3)	5	P12763	FETUA_BOVIN
1634.2060	VVHAVEVALATFNAESNGSYLQLVEISR	HexNAc(6)Hex(7)NeuAc(4)	4	P12763	FETUA_BOVIN
1029.4569	VVHAVEVALATFNAESNGSYLQLVEISR	HexNAc(7)Hex(8)Fuc(1)NeuAc(1)	6	P12763	FETUA_BOVIN

Table S3. The inclusion list of glycopeptides for HeLa samples according to *m*/*z*, peptide sequence, glycan composition, charge, protein ID and accession number.

m/z	Peptide sequence	Glycan composition	Charge	Accession number	Protein ID
1026.0675	VSNYSR	HexNAc(4)Hex(5)Fuc(1)NeuAc(2)	3	P15328	FOLR1_HUMAN
990.3765	NACCSTNTSQEAHK	HexNAc(4)Hex(5)Fuc(1)NeuAc(2)	4	P15328	FOLR1_HUMAN
1277.0076	AANGSLR	HexNAc(2)Hex(9)	2	P11279	LAMP1_HUMAN
1195.9811	AANGSLR	HexNAc(2)Hex(8)	2	P11279	LAMP1_HUMAN
1269.5076	SLSNSTAR	HexNAc(2)Hex(8)	2	P50454	SERPH_HUMAN
1107.4551	SLSNSTAR	HexNAc(2)Hex(6)	2	P50454	SERPH_HUMAN
1324.5441	NATLAEQAK	HexNAc(2)Hex(8)	2	Q9Y4L1	HYOU1_HUMAN
1427.5311	NYTADYDK	HexNAc(2)Hex(9)	2	O94905	ERLN2_HUMAN
898.0055	NYTADYDK	HexNAc(2)Hex(8)	3	O94905	ERLN2_HUMAN
1346.5046	NYTADYDK	HexNAc(2)Hex(8)	2	O94905	ERLN2_HUMAN
923.7092	YIHQNYTK	HexNAc(2)Hex(8)	3	Q02809	PLOD1_HUMAN
1173.7549	CHEGNGTFECGACR	HexNAc(2)Hex(9)	3	P05556	ITB1_HUMAN
886.3775	HLNGTITAK	HexNAc(2)Hex(8)	3	Q6YHK3	CD109_HUMAN
1138.7825	ENGTDTVQEEEESPAEGSK	HexNAc(2)Hex(6)	3	Q9Y4L1	HYOU1_HUMAN
990.7052	MNFTGGDTCHK	HexNAc(2)Hex(8)	3	P11717	MPRI_HUMAN
948.3905	NNHTASILDR	HexNAc(2)Hex(8)	3	P08962	CD63_HUMAN
848.6992	NNHTASILDR	HexNAc(3)Hex(4)Fuc(1)	3	P08962	CD63_HUMAN
957.7042	YHYNGTFEDGK	HexNAc(2)Hex(7)	3	Q96AY3	FKB10_HUMAN
903.6869	YHYNGTFEDGK	HexNAc(2)Hex(6)	3	Q96AY3	FKB10_HUMAN
849.6692	YHYNGTFEDGK	HexNAc(2)Hex(5)	3	Q96AY3	FKB10_HUMAN
886.7145	VQPFNVTQGK	HexNAc(2)Hex(7)	3	P13473	LAMP2_HUMAN
1020.4442	LLNINPNK	HexNAc(5)Hex(6)Fuc(1)	3	P11279	LAMP1_HUMAN
900.3899	VQPFNVTQGK	HexNAc(3)Hex(6)	3	P13473	LAMP2_HUMAN
1233.5411	LLNINPNK	HexNAc(2)Hex(7)	2	P11279	LAMP1_HUMAN
898.7335	LLNINPNK	HexNAc(4)Hex(5)Fuc(1)	3	P11279	LAMP1_HUMAN
1071.4881	LLNINPNK	HexNAc(2)Hex(5)	2	P11279	LAMP1_HUMAN
900.7215	GHTLTLNFTR	HexNAc(2)Hex(7)	3	P11279	LAMP1_HUMAN
846.7042	GHTLTLNFTR	HexNAc(2)Hex(6)	3	P11279	LAMP1_HUMAN
1200.8492	TILVDNNTWNNTHISR	HexNAc(2)Hex(8)	3	P46977	STT3A_HUMAN
920.0492	NVSTNVFFK	HexNAc(2)Hex(8)	3	Q6YHK3	CD109_HUMAN
1011.4289	GHTLTLNFTR	HexNAc(3)Hex(6)NeuAc(1)	3	P11279	LAMP1_HUMAN
1083.4322	VNFTLEASEGCYR	HexNAc(2)Hex(8)	3	Q8TEM1	PO210_HUMAN
1624.6446	VNFTLEASEGCYR	HexNAc(2)Hex(8)	2	Q8TEM1	PO210_HUMAN
1092.4415	QNNGAFNETLFR	HexNAc(2)Hex(9)	3	P02786	TFR1_HUMAN
1038.4239	QNNGAFNETLFR	HexNAc(2)Hex(8)	3	P02786	TFR1_HUMAN
1425.0916	VINETWAWK	HexNAc(2)Hex(8)	2	Q9Y4L1	HYOU1_HUMAN
1291.1909	YHYNGTLLDGTSFDTSYSK	HexNAc(2)Hex(8)	3	Q96AY3	FKB10_HUMAN
1133.7972	NMSFVNDLTVTQDGR	HexNAc(2)Hex(8)	3	Q9HDC9	APMAP_HUMAN
1025.7622	NMSFVNDLTVTQDGR	HexNAc(2)Hex(6)	3	Q9HDC9	APMAP_HUMAN
1104.2027	HNNDTQHIWESDSNEFSVIADPR	HexNAc(2)Hex(8)	4	P14625	ENPL_HUMAN
1023.1765	HNNDTQHIWESDSNEFSVIADPR	HexNAc(2)Hex(6)	4	P14625	ENPL_HUMAN
1278.1559	GSQWSDIEEFCNR	HexNAc(4)Hex(5)NeuAc(2)	3	P08174	DAF_HUMAN
1305.8522	YHYNGSLMDGTLFDSSYSR	HexNAc(2)Hex(8)	3	Q96AY3	FKB10_HUMAN

1067.4589	ENTSDPSLVIAFGR	HexNAc(3)Hex(4)Fuc(1)NeuAc(1)	3	P11279	LAMP1_HUMAN
1100.4549	DMSDGFISNLTIQR	HexNAc(2)Hex(8)	3	P13674	P4HA1_HUMAN
1304.8419	GWNWTSGFNK	HexNAc(5)Hex(6)Fuc(1)NeuAc(2)	3	P15328	FOLR1_HUMAN
1134.4452	GWNWTSGFNK	HexNAc(4)Hex(5)NeuAc(2)	3	P15328	FOLR1_HUMAN
1180.7297	QQQHLFGSNVTDCSGNFCLFR	HexNAc(4)Hex(5)NeuAc(2)	3	P02787	TRFE_HUMAN
1332.9169	DFEDLYTPVNGSIVIVR	HexNAc(4)Hex(5)Fuc(1)NeuAc(1)	3	P02786	TFR1_HUMAN

Table S4. The inclusion list of glycopeptides for blood plasma samples according to *m*/*z*, peptide sequence, glycan composition, charge, protein ID and accession number.

m*/*z	**Peptide sequence**	**Glycan composition**	**Charge**	**Accession number**	**Protein ID**
1074.1597	ENGTISR	HexNAc(6)Hex(7)NeuAc(4)	4	P02763	A1AG1_HUMAN
951.6067	NEEYNK	HexNAc(5)Hex(6)Fuc(1)NeuAc(3)	4	P02763	A1AG1_HUMAN
1268.4732	NEEYNK	HexNAc(5)Hex(6)Fuc(1)NeuAc(3)	3	P02763	A1AG1_HUMAN
915.0922	NEEYNK	HexNAc(5)Hex(6)NeuAc(3)	4	P02763	A1AG1_HUMAN
1219.7872	NEEYNK	HexNAc(5)Hex(6)NeuAc(3)	3	P02763	A1AG1_HUMAN
1122.7555	NEEYNK	HexNAc(5)Hex(6)NeuAc(2)	3	P02763	A1AG1_HUMAN
842.3185	NEEYNK	HexNAc(5)Hex(6)NeuAc(2)	4	P02763	A1AG1_HUMAN
1001.0449	NEEYNK	HexNAc(4)Hex(5)NeuAc(2)	3	P02763	A1AG1_HUMAN
751.0355	NEEYNK	HexNAc(4)Hex(5)NeuAc(2)	4	P02763	A1AG1_HUMAN
982.7095	SYNETK	HexNAc(4)Hex(5)NeuAc(2)	3	P04114	APOB_HUMAN
1031.7469	NVTAEQAR	HexNAc(4)Hex(5)NeuAc(2)	3	P00748	FA12_HUMAN
836.8437	YKNNSDISSTR	HexNAc(4)Hex(5)Fuc(1)NeuAc(1)	4	P01871	IGHM_HUMAN
1115.4559	YKNNSDISSTR	HexNAc(4)Hex(5)Fuc(1)NeuAc(1)	3	P01871	IGHM_HUMAN
1018.4239	YKNNSDISSTR	HexNAc(4)Hex(5)Fuc(1)	3	P01871	IGHM_HUMAN
1083.7539	EEQYNSTYR	HexNAc(4)Hex(5)Fuc(1)NeuAc(1)	3	P01857	IGHG1_HUMAN
813.0672	EEQYNSTYR	HexNAc(4)Hex(5)Fuc(1)NeuAc(1)	4	P01857	IGHG1_HUMAN
1001.0765	GHVNITR	HexNAc(4)Hex(5)NeuAc(2)	3	P00734	THRB_HUMAN
986.7222	EEQYNSTYR	HexNAc(4)Hex(5)Fuc(1)	3	P01857	IGHG1_HUMAN
740.2935	EEQYNSTYR	HexNAc(4)Hex(5)Fuc(1)	4	P01857	IGHG1_HUMAN
1479.5796	EEQYNSTYR	HexNAc(4)Hex(5)Fuc(1)	2	P01857	IGHG1_HUMAN
938.0362	EEQYNSTYR	HexNAc(4)Hex(5)	3	P01857	IGHG1_HUMAN
904.0445	GHVNITR	HexNAc(4)Hex(5)NeuAc(1)	3	P00734	THRB_HUMAN
933.6415	TKPREEQYNSTYR	HexNAc(4)Hex(5)Fuc(1)NeuAc(1)	4	P01857	IGHG1_HUMAN
860.8675	TKPREEQYNSTYR	HexNAc(4)Hex(5)Fuc(1)	4	P01857	IGHG1_HUMAN
1075.7639	FLNNGTCTAEGK	HexNAc(4)Hex(5)NeuAc(1)	3	P05156	CFAI_HUMAN
981.3905	EEQFNSTYR	HexNAc(4)Hex(5)Fuc(1)	3	P01861	IGHG4_HUMAN
852.8700	TKPREEQFNSTFR	HexNAc(4)Hex(5)Fuc(1)	4	P01859	IGHG2_HUMAN
1128.1485	VYKPSAGNNSLYR	HexNAc(4)Hex(5)NeuAc(1)	3	P02749	APOH_HUMAN
919.1372	VYKPSAGNNSLYR	HexNAc(4)Hex(5)NeuAc(2)	4	P02749	APOH_HUMAN
955.6517	VYKPSAGNNSLYR	HexNAc(4)Hex(5)Fuc(1)NeuAc(2)	4	P02749	APOH_HUMAN
976.0589	EEQFNSTFR	HexNAc(4)Hex(5)Fuc(1)	3	P01859	IGHG2_HUMAN
1463.5846	EEQFNSTFR	HexNAc(4)Hex(5)Fuc(1)	2	P01859	IGHG2_HUMAN

732.2960	EEQFNSTFR	HexNAc(4)Hex(5)Fuc(1)	4	P01859	IGHG2_HUMAN
1006.7689	TPLTANITK	HexNAc(4)Hex(5)Fuc(1)NeuAc(1)	3	P01877	IGHA2_HUMAN
1073.0905	EEQFNSTFR	HexNAc(4)Hex(5)Fuc(1)NeuAc(1)	3	P01859	IGHG2_HUMAN
805.0697	EEQFNSTFR	HexNAc(4)Hex(5)Fuc(1)NeuAc(1)	4	P01859	IGHG2_HUMAN
988.8995	EHEGAIYPDNTTDFQR	HexNAc(4)Hex(5)Fuc(1)NeuAc(1)	4	P00450	CERU_HUMAN
1048.1029	ENISDPTSPLR	HexNAc(4)Hex(5)NeuAc(1)	3	P01591	IGJ_HUMAN
952.3850	EHEGAIYPDNTTDFQR	HexNAc(4)Hex(5)NeuAc(1)	4	P00450	CERU_HUMAN
1061.6732	EHEGAIYPDNTTDFQR	HexNAc(4)Hex(5)Fuc(1)NeuAc(2)	4	P00450	CERU_HUMAN
1025.1587	EHEGAIYPDNTTDFQR	HexNAc(4)Hex(5)NeuAc(2)	4	P00450	CERU_HUMAN
1009.7529	VLNFTTK	HexNAc(4)Hex(5)NeuAc(2)	3	P13671	CO6_HUMAN
996.6577	ITYSIVQTNCSK	HexNAc(5)Hex(6)NeuAc(2)	4	P01042	KNG1_HUMAN
1055.4192	LGNWSAMPSCK	HexNAc(4)Hex(5)NeuAc(1)	3	P02749	APOH_HUMAN
1213.8205	FSDGLESNSSTQFEVK	HexNAc(2)Hex(9)	3	P0C0L5	CO4B_HUMAN
864.5900	LGNWSAMPSCK	HexNAc(4)Hex(5)NeuAc(2)	4	P02749	APOH_HUMAN
1152.4509	LGNWSAMPSCK	HexNAc(4)Hex(5)NeuAc(2)	3	P02749	APOH_HUMAN
900.6307	FNSSYLQGTNQITGR	HexNAc(4)Hex(5)NeuAc(1)	4	P04114	APOB_HUMAN
1200.5052	FNSSYLQGTNQITGR	HexNAc(4)Hex(5)NeuAc(1)	3	P04114	APOB_HUMAN
847.1105	HANWTLTPLK	HexNAc(4)Hex(5)NeuAc(2)	4	P27169	PON1_HUMAN
949.6777	TVLTPATNHMGNVTFTIPANR	HexNAc(2)Hex(7)	4	P01024	CO3_HUMAN
1265.9012	TVLTPATNHMGNVTFTIPANR	HexNAc(2)Hex(7)	3	P01024	CO3_HUMAN
1211.8835	TVLTPATNHMGNVTFTIPANR	HexNAc(2)Hex(6)	3	P01024	CO3_HUMAN
909.1645	TVLTPATNHMGNVTFTIPANR	HexNAc(2)Hex(6)	4	P01024	CO3_HUMAN
1130.8145	CGLVPVLAENYNK	HexNAc(4)Hex(5)NeuAc(1)	3	P02787	TRFE_HUMAN
1136.7175	GLTFQQNASSMCVPDQDTAIR	HexNAc(4)Hex(5)NeuAc(2)	4	P01871	IGHM_HUMAN
1229.1795	VCQDCPLLAPLNDTR	HexNAc(4)Hex(5)NeuAc(1)	3	P02765	FETUA_HUMAN
922.1365	VCQDCPLLAPLNDTR	HexNAc(4)Hex(5)NeuAc(1)	4	P02765	FETUA_HUMAN
1019.2107	VVLHPNYSQVDIGLIK	HexNAc(5)Hex(6)NeuAc(1)	4	P00738	HPT_HUMAN
927.9275	VVLHPNYSQVDIGLIK	HexNAc(4)Hex(5)NeuAc(1)	4	P00738	HPT_HUMAN
921.1365	CGLVPVLAENYNK	HexNAc(4)Hex(5)NeuAc(2)	4	P02787	TRFE_HUMAN
1227.8462	CGLVPVLAENYNK	HexNAc(4)Hex(5)NeuAc(2)	3	P02787	TRFE_HUMAN
1236.9009	VVLHPNYSQVDIGLIK	HexNAc(4)Hex(5)NeuAc(1)	3	P00738	HPT_HUMAN
1076.2490	MVSHHNLTTGATLINEQWLLTTAK	HexNAc(4)Hex(5)	4	P00738	HPT_HUMAN
1070.4630	AALAAFNAQNNGSNFQLEEISR	HexNAc(4)Hex(5)NeuAc(1)	4	P02765	FETUA_HUMAN
1091.9845	VVLHPNYSQVDIGLIK	HexNAc(5)Hex(6)NeuAc(2)	4	P00738	HPT_HUMAN
1333.9329	VVLHPNYSQVDIGLIK	HexNAc(4)Hex(5)NeuAc(2)	3	P00738	HPT_HUMAN
1000.7015	VVLHPNYSQVDIGLIK	HexNAc(4)Hex(5)NeuAc(2)	4	P00738	HPT_HUMAN
1149.0227	MVSHHNLTTGATLINEQWLLTTAK	HexNAc(4)Hex(5)NeuAc(1)	4	P00738	HPT_HUMAN
919.4196	MVSHHNLTTGATLINEQWLLTTAK	HexNAc(4)Hex(5)NeuAc(1)	5	P00738	HPT_HUMAN
1314.2222	ALPQPQNVTSLLGCTH	HexNAc(4)Hex(5)NeuAc(2)	3	P02790	HEMO_HUMAN
985.9185	ALPQPQNVTSLLGCTH	HexNAc(4)Hex(5)NeuAc(2)	4	P02790	HEMO_HUMAN
977.6388	MVSHHNLTTGATLINEQWLLTTAK	HexNAc(4)Hex(5)NeuAc(2)	5	P00738	HPT_HUMAN
1221.7967	MVSHHNLTTGATLINEQWLLTTAK	HexNAc(4)Hex(5)NeuAc(2)	4	P00738	HPT_HUMAN
1180.7297	QQQHLFGSNVTDCSGNFCLFR	HexNAc(4)Hex(5)NeuAc(2)	4	P02787	TRFE_HUMAN

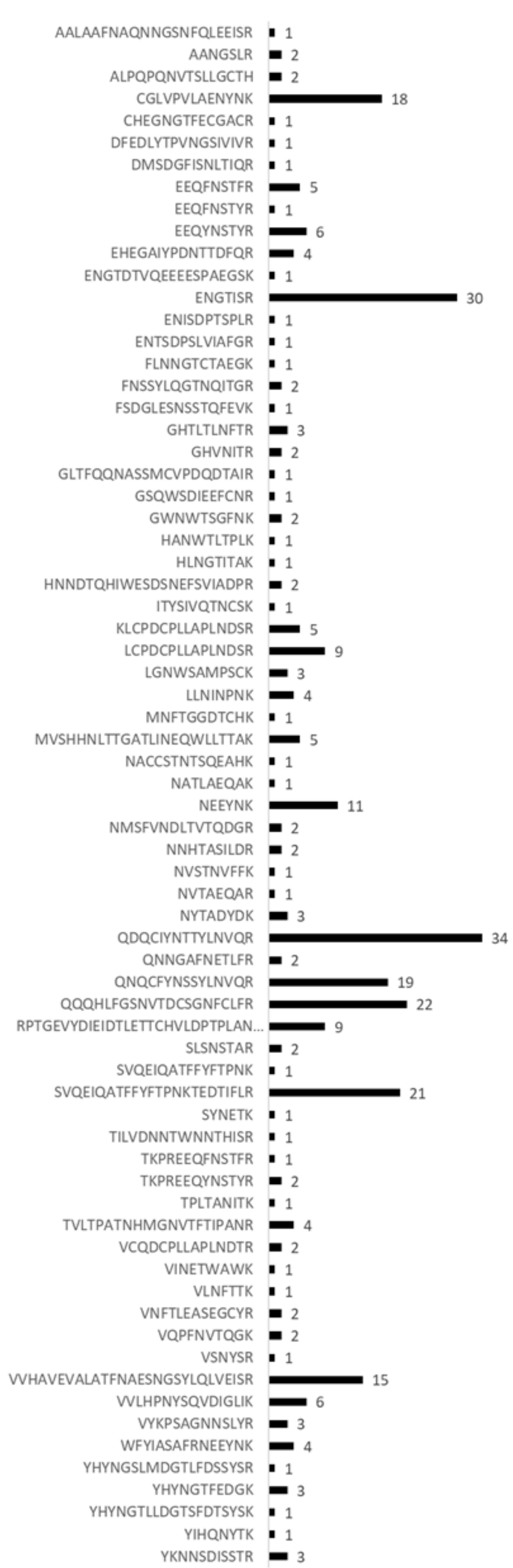

Sample	Glycans strusture	Charge state
std mix 1th	HexNAc(4)Hex(5)NeuAc(2)	4
std mix 1th	HexNAc(5)Hex(6)Fuc(1)NeuAc(2)	4
std mix 1th	HexNAc(5)Hex(6)Fuc(1)NeuAc(3)	4
std mix 1th	HexNAc(5)Hex(6)Fuc(1)NeuAc(3)	5
std mix 1th	HexNAc(5)Hex(6)NeuAc(1)	4
std mix 1th	HexNAc(5)Hex(6)NeuAc(2)	4
std mix 1th	HexNAc(5)Hex(6)NeuAc(2)	5
std mix 1th	HexNAc(5)Hex(6)NeuAc(2)	3
std mix 1th	HexNAc(5)Hex(6)NeuAc(3)	4
std mix 1th	HexNAc(5)Hex(6)NeuAc(3)	3
std mix 1th	HexNAc(5)Hex(6)NeuAc(3)	5
std mix 1th	HexNAc(6)Hex(7)Fuc(1)NeuAc(1)	4
std mix 1th	HexNAc(6)Hex(7)Fuc(1)NeuAc(2)	4
std mix 1th	HexNAc(6)Hex(7)Fuc(1)NeuAc(2)	5
std mix 2nd	HexNAc(6)Hex(7)Fuc(1)NeuAc(2)	5
std mix 1th	HexNAc(6)Hex(7)Fuc(1)NeuAc(3)	4
std mix 1th	HexNAc(6)Hex(7)Fuc(1)NeuAc(3)	5
std mix 1th	HexNAc(6)Hex(7)Fuc(1)NeuAc(4)	4
std mix 1th	HexNAc(6)Hex(7)NeuAc(1)	5
std mix 1th	HexNAc(6)Hex(7)NeuAc(1)	4
std mix 2nd	HexNAc(6)Hex(7)NeuAc(1)	5
std mix 1th	HexNAc(6)Hex(7)NeuAc(2)	4
std mix 1th	HexNAc(6)Hex(7)NeuAc(2)	5
std mix 1th	HexNAc(6)Hex(7)NeuAc(2)	3
std mix 1th	HexNAc(6)Hex(7)NeuAc(3)	5
std mix 1th	HexNAc(6)Hex(7)NeuAc(3)	4
std mix 1th	HexNAc(6)Hex(7)NeuAc(3)	3
std mix 1th	HexNAc(6)Hex(7)NeuAc(4)	4
std mix 1th	HexNAc(6)Hex(7)NeuAc(4)	5
std mix 1th	HexNAc(7)Hex(8)Fuc(1)NeuAc(4)	4
std mix 2nd	HexNAc(7)Hex(8)Fuc(1)NeuAc(4)	4
std mix 1th	HexNAc(7)Hex(8)NeuAc(1)	4
std mix 2nd	HexNAc(7)Hex(8)NeuAc(1)	4
std mix 1th	HexNAc(8)Hex(9)Fuc(1)	5

Fig. S1. The Number of N-glycopeptide Species as a Function of Peptide Structure. On the left side: graphical representation of the number of all species with: (1) different glycan structures, (2) different charge levels, and (3) from different series of measurements (nLC-(nESI)MS/MS runs) as a function of peptide sequence, highlighted by the inclusion lists in the optimal CE study. On the right side: single highlighted peptide sequence (QDQCIYNTTYLNVQR) with all 34 identified species: (1) with different glycan structures, (2) with different charge levels and (3) from different measurement sequences.

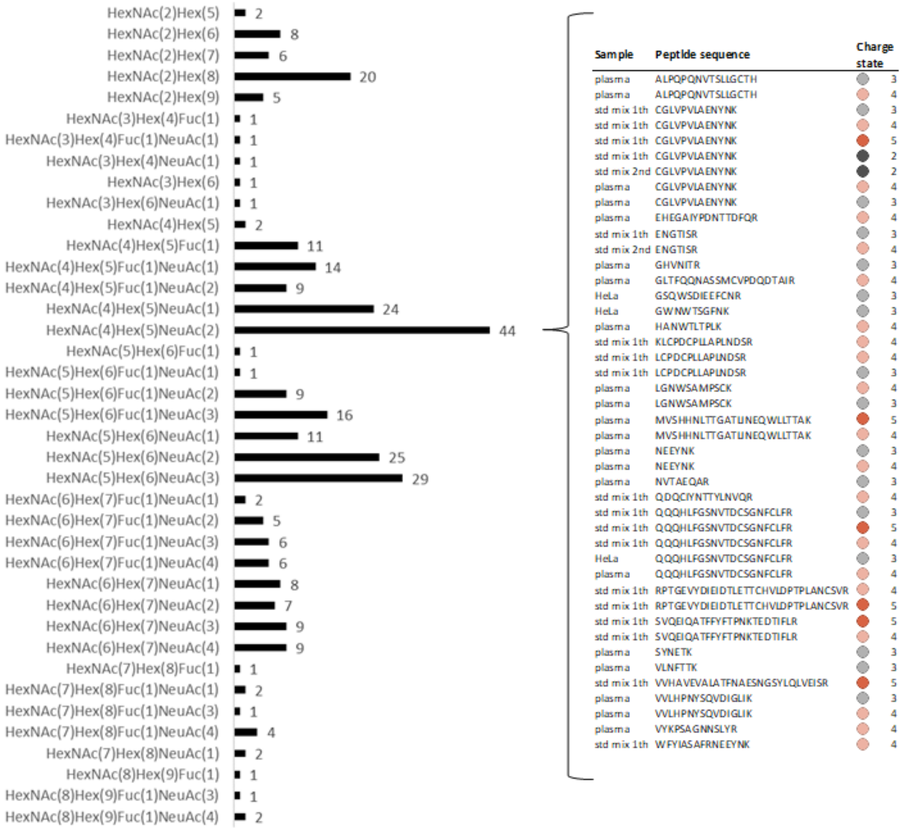

Fig. S2. The Number of N-glycopeptide Species as a Function of Glycan Structure. On the left side: graphical representation of the number of species with: (1) different peptide sequences, (2) different charge levels and (3) from different measurement sequences (nLC-(nESI)MS/MS runs) as a function of glycan structures, highlighted by the inclusion lists of the optimal CE study. On the right side: single highlighted N-glycan structure (HexNAc(4)Hex(5)NeuAc(2)) with all 44 identified species: (1) with different peptide sequences, (2) with different charge levels and (3) from different measurement sequences.

Material S3: *N*-glycopeptide Identification Using Various Search Engines

Byonic

The measurements of glycoprotein mixture were evaluated using the amino acid sequences of the 3 glycoprotein standards (obtained from UniProt, December 2019); human SwissProt (November 2020) database was applied for the analysis of blood plasma experiments, while a focused database obtained from human SwissProt database based on previous HeLa experiments was used for HeLa samples. Byonic searches were carried out with the human N-glycan database of 182 structures without multiple fucose as implemented in Byonic. Trypsin was set as enzyme; a maximum of two missed cleavages were allowed, and cysteine carbamidomethylation was selected as a fixed modification. Regarding mass tolerance values, recommendations of the Preview module of Byonic were used. The Byonic Excel reports were the input files for data aggregation carried out by the Serac program in the energy dependent studies.

pGlyco

In all cases, the same protein databases were used pGlyco and Byonic search engine. The pGlyco.gdb N-glycan database was applied as glycan database. All other parameters were set equivalent to the Byonic search and pGlyco FDR-Pro.txt reports were the input files for further analysis.

GlycoQuest

GlycoQuest was used as integrated in ProteinScape. Both H+ and HexNAc+ classification were applied and hits from the two searches were merged. GlycomDB was used as glycan database, N-glycan was set as glycan type and composition was restricted to Hexose(4-10), N-Acetylglucosamine(1-8), Sialic acid(0-4) and Fucose(0-1). Precursor and fragment mass tolerance was set to 15 ppm and 0.05 Da, respectively. Excel reports obtained from ProteinScape were

used for energy dependence investigations. Only hits having reported peptide mass that could be correlated to peptide sequence identified with Byonic and/or pGlyco were further analyzed.

Material S4: Determination of Optimal CE setting Using Serac

First, score data were extracted from the Byonic Excel reports, the FDR-Pro.txt output files of the pGlyco program and Excel reports of the GlycoQuest results. Then, the Serac program normalized the score vs. CE setting functions by dividing all values with the maximum score for the given glycopeptide ion.

First, depending on the chosen measure of identification confidence, Serac program only considered an *N*-glycopeptide ion identified at a given CE setting if its Byonic score exceeded 100, or its pGlyco score was above 5. Further, a glycopeptide ion was only included in the energy-dependence analysis if it was identified at least at six consecutive collision energy settings and for at least one collision energy it was found to have a Byonic score value above 300 (being a "good" score), or the investigated pGlyco score above 15, or GlycoQuest score above 30. Finally, due to the significant tailing of GlycoQuest energy dependent curves to the high CE direction, identifications below the 40% of the maximum score of the given *N*-glycopeptide were ignored,

For each *N*-glycopeptide, the Serac program determined the optimum energy from the normalized score vs. collision energy setting data sets by fitting Gaussian functions. The score cutoff, while important to avoid false identifications biasing our results, resulted in no data points at low scores; therefore, two additional points with zero score at extremely low and extremely large CE settings were added to avoid erroneously wide peaks to be fitted. The nonlinear fits were carried out by Serac, and the corresponding plots were generated using the levmar and PGPLOT libraries through their Perl Data Language interfaces. The positions of the center of the Gaussian peaks were considered as optimal values. The optimal CE data of the five different scores were subject to statistical analysis.

Table S5: Details of Statistical Methods. The results presented in the paper are based on fitting three different GLMs to our datasets. Each model fitting involved optimal CEs for one specific search engine score, and was repeated for all four studied scores, so altogether we carried out 12 fits.

Case	Dependent variable	Independent variables	Comments
**Hydrophobicity (Figure 2)**	Optimal CE	*m/z* (continuous), hydrophobicity (continuous)	Reported *p* values refer to statistical significance of the coefficient of hydrophobicity being nonzero.
**Number of mobile protons (Figure 3)**	Optimal CE	*m/z* (continuous), number of mobile protons (categorical)	Zero mobile protons were considered the baseline. Reported *p* values refer to statistical significance of the intercept corresponding to a given number of mobile protons being different from that of the zero mobile protons.
**Number of sialic acid units (Figure 4)**	Optimal CE	*m/z* (continuous), number of sialic acid units (categorical)	Zero sialic acid units were considered the baseline. Reported *p* values refer to statistical significance of the intercept corresponding to a given number of sialic acid units being different from that of the zero sialic acid units.

Material S5: Variables Used in Lasso Regression

The following glycopeptide parameters were analyzed in lasso regression: charge, *m/z*, hydrophobicity, gravy index score, isoelectric point, peptide length, total neutral mass, neutral mass of glycan part, number of mobile protons (expressed as charge – Lys – Arg – 1), number of mobile protons (expressed as charge – Lys – Arg – His – 1), peptide *m/z* (expressed as (peptide neutral mass + charge)/charge), numbers of HexNAc/hexose/fucose/NeuAc units, numbers of all amino acids individually, numbers of Lys and Arg combined, numbers of His, Arg, Lys combined, position of the glycan-bearing Asn amino acid (1,2,3…), the same position expressed as a fraction of peptide length.

N-glycopeptide Level Optimal Collision Energy

Byonic	●	○	○	○	●
pGlyco Peptide	○	●	○	○	●
pGlyco Glycan	○	○	●	○	○
GlycoQuest	○	○	○	●	○
# of unique N-glycopeptid structures considered in CE study	**35**	**0**	**1**	**13**	**2**
	AALAAFNAQNNGSNFQLEEISR-HexNAc(4)Hex(5)NeuAc(1)	-	IERSFLDCTNR-HexNAc(2)Hex(8)	ENGTISR-HexNAc(6)Hex(7)Fuc(1)NeuAc(1)	QDQCIYNTTYLNVQR-HexNAc(5)Hex(6)NeuAc(1)
	CGLVPVLAENYNK-HexNAc(4)Hex(5)Fuc(1)NeuAc(1)			ENISDPTSPLR-HexNAc(4)Hex(5)NeuAc(1)	YHYNGTFEDGK-HexNAc(2)Hex(7)
	CHEGNGTFECGACR-HexNAc(2)Hex(9)			GHVNITR-HexNAc(4)Hex(5)NeuAc(1)	
	EEQYNSTYR-HexNAc(4)Hex(4)Fuc(1)			GHVNITR-HexNAc(4)Hex(5)NeuAc(2)	
	EHEGAIYPDNTTDFQR-HexNAc(4)Hex(5)Fuc(1)NeuAc(1)			NNHTASILDR-HexNAc(2)Hex(8)	
	EHEGAIYPDNTTDFQR-HexNAc(4)Hex(5)Fuc(1)NeuAc(2)			NVTAEQAR-HexNAc(4)Hex(5)NeuAc(2)	
	EHEGAIYPDNTTDFQR-HexNAc(4)Hex(5)NeuAc(1)			QNNGAFNETLFR-HexNAc(2)Hex(8)	
	ENGTISR-HexNAc(8)Hex(9)Fuc(1)NeuAc(3)			QNNGAFNETLFR-HexNAc(2)Hex(9)	
	ENGTVSR-HexNAc(5)Hex(6)NeuAc(2)			SLSNSTAR-HexNAc(2)Hex(6)	
	HNNDTQHIWESDSNEFSVIADPR-HexNAc(2)Hex(6)			SLSNSTAR-HexNAc(2)Hex(8)	
	LLNINPNK-HexNAc(2)Hex(7)			SVQEIQATFFYFTPNKTEDTIFLR-HexNAc(5)Hex(6)Fuc(1)NeuAc(2)	
	MVSHHNLTTGATLINEQWLLTTAK-HexNAc(4)Hex(5)			VLNFTTK-HexNAc(4)Hex(5)NeuAc(2)	
	NMSFVNDLTVTQDGR-HexNAc(2)Hex(6)			VYKPSAGNNSLYR-HexNAc(4)Hex(5)NeuAc(1)	
	NMSFVNDLTVTQDGR-HexNAc(2)Hex(8)				
	QDQCIYNTTYLNVQR-HexNAc(6)Hex(7)Fuc(1)NeuAc(1)				
	QDQCIYNTTYLNVQR-HexNAc(7)Hex(8)Fuc(1)				
	QDQCIYNTTYLNVQR-HexNAc(7)Hex(8)NeuAc(1)				
	QNQCFYNSSYLNVQR-HexNAc(5)Hex(6)Fuc(1)NeuAc(2)				
	QNQCFYNSSYLNVQR-HexNAc(5)Hex(6)NeuAc(1)				
	QNQCFYNSSYLNVQR-HexNAc(6)Hex(7)Fuc(1)NeuAc(2)				
	QQQHLFGSNVTDCSGNFCLFR-HexNAc(3)Hex(4)NeuAc(1)				
	QQQHLFGSNVTDCSGNFCLFR-HexNAc(5)Hex(6)NeuAc(2)				
	SVQEIQATFFYFTPNKTEDTIFLR-HexNAc(4)Hex(5)NeuAc(1)				
	SVQEIQATFFYFTPNKTEDTIFLR-HexNAc(5)Hex(6)NeuAc(1)				
	SVQEIQATFFYFTPNKTEDTIFLR-HexNAc(6)Hex(7)Fuc(1)NeuAc(2)				
	SVQEIQATFFYFTPNKTEDTIFLR-HexNAc(6)Hex(7)Fuc(1)NeuAc(4)				
	SVQEIQATFFYFTPNKTEDTIFLR-HexNAc(6)Hex(7)NeuAc(1)				
	TVLTPATNHMGNVTFTIPANR-HexNAc(2)Hex(7)				
	VINETWAWK-HexNAc(2)Hex(8)				
	VNFTLEASEGCYR-HexNAc(2)Hex(8)				
	VQPFNVTQGK-HexNAc(3)Hex(6)				
	VVHAVEVALATFNAESNGSYLQLVEISR-HexNAc(5)Hex(6)Fuc(1)NeuAc(3)				
	VVLHPNYSQVDIGLIK-HexNAc(5)Hex(6)NeuAc(1)				
	VVLHPNYSQVDIGLIK-HexNAc(5)Hex(6)NeuAc(2)				
	YHYNGTFEDGK-HexNAc(2)Hex(5)				

Byonic	●	●	○	○	○
pGlyco Peptide	○	○	●	●	○
pGlyco Glycan	●	○	●	○	●
GlycoQuest	○	●	○	●	●
# of unique N-glycopeptid structures considered in CE study	**3**	**39**	**1**	**0**	**0**
	SVQEIQATFFYFTPNKTEDTIFLR-HexNAc(6)Hex(7)NeuAc(2)<br>SVQEIQATFFYFTPNKTEDTIFLR-HexNAc(6)Hex(7)NeuAc(3)<br>YHYNGSLMDGTLFDSSYSR-HexNAc(2)Hex(8)	CGLVPVLAENYNK-HexNAc(4)Hex(5)Fuc(1)NeuAc(2)<br>EEQFNSTFR-HexNAc(4)Hex(5)Fuc(1)NeuAc(1)<br>EEQFNSTYR-HexNAc(4)Hex(5)Fuc(1)<br>EEQYNSTYR-HexNAc(4)Hex(5)<br>EEQYNSTYR-HexNAc(4)Hex(5)Fuc(1)<br>EEQYNSTYR-HexNAc(4)Hex(5)Fuc(1)NeuAc(1)<br>EHEGAIYPDNTTDFQR-HexNAc(4)Hex(5)NeuAc(2)<br>ENTSDPSLVIAFGR-HexNAc(3)Hex(4)Fuc(1)NeuAc(1)<br>FNSSYLQGTNQITGR-HexNAc(4)Hex(5)NeuAc(1)<br>GHTLTLNFTR-HexNAc(3)Hex(6)NeuAc(1)<br>GWNWTSGFNK-HexNAc(4)Hex(5)NeuAc(2)<br>GWNWTSGFNK-HexNAc(5)Hex(6)Fuc(1)NeuAc(2)<br>HANWTLTPLK-HexNAc(4)Hex(5)NeuAc(2)<br>HNNDTQHIWESDSNEFSVIADPR-HexNAc(2)Hex(8)<br>LGNWSAMPSCK-HexNAc(4)Hex(5)NeuAc(1)<br>MVSHHNLTTGATLINEQWLLTTAK-HexNAc(4)Hex(5)NeuAc(1)<br>MVSHHNLTTGATLINEQWLLTTAK-HexNAc(4)Hex(5)NeuAc(2)<br>NEEYNK-HexNAc(5)Hex(6)Fuc(1)NeuAc(3)<br>NEEYNK-HexNAc(5)Hex(6)NeuAc(1)<br>NVSTNVFFK-HexNAc(2)Hex(8)<br>NYTADYDK-HexNAc(2)Hex(9)<br>QNQCFYNSSYLNVQR-HexNAc(6)Hex(7)Fuc(1)NeuAc(3)<br>QQQHLFGSNVTDCSGNFCLFR-HexNAc(4)Hex(5)Fuc(1)NeuAc(1)<br>QQQHLFGSNVTDCSGNFCLFR-HexNAc(4)Hex(5)NeuAc(1)<br>SVQEIQATFFYFTPNKTEDTIFLR-HexNAc(5)Hex(6)Fuc(1)NeuAc(3)<br>TKPREEQFNSTFR-HexNAc(4)Hex(5)Fuc(1)<br>TKPREEQYNSTYR-HexNAc(4)Hex(5)Fuc(1)<br>TKPREEQYNSTYR-HexNAc(4)Hex(5)Fuc(1)NeuAc(1)<br>TPLTANITK-HexNAc(4)Hex(5)Fuc(1)NeuAc(1)<br>TVLTPATNHMGNVTFTIPANR-HexNAc(2)Hex(6)<br>VCQDCPLLAPLNDTR-HexNAc(4)Hex(5)NeuAc(1)<br>VQPFNVTQGK-HexNAc(2)Hex(7)<br>VVHAVEVALATFNAESNGSYLQLVEISR-HexNAc(5)Hex(6)NeuAc(1)<br>VVLHPNYSQVDIGLIK-HexNAc(4)Hex(5)NeuAc(1)<br>VVLHPNYSQVDIGLIK-HexNAc(4)Hex(5)NeuAc(2)<br>VYKPSAGNNSLYR-HexNAc(4)Hex(5)Fuc(1)NeuAc(2)<br>VYKPSAGNNSLYR-HexNAc(4)Hex(5)NeuAc(2)<br>YKNNSDISSTR-HexNAc(4)Hex(5)Fuc(1)<br>YKNNSDISSTR-HexNAc(4)Hex(5)Fuc(1)NeuAc(1)	VVHAVEVALATFNAESNGSYLQLVEISR-HexNAc(5)Hex(6)NeuAc(4)	-	-

Byonic	●	●	●	○	●
pGlyco Peptide	●	●	○	●	●
pGlyco Glycan	●	○	●	●	●
GlycoQuest	○	●	●	●	●
# of unique N-glycopeptid structures considered in CE study	**14**	**3**	**17**	**0**	**56**
	ENGTDTVQEEEESPAEGSK-HexNAc(2)Hex(6)<br>ENGTISR-HexNAc(6)Hex(7)Fuc(1)NeuAc(2)<br>ENGTISR-HexNAc(6)Hex(7)NeuAc(2)<br>ENGTISR-HexNAc(6)Hex(7)NeuAc(3)<br>ENGTISR-HexNAc(7)Hex(8)Fuc(1)NeuAc(3)<br>QDQCIYNTTYLNVQR-HexNAc(5)Hex(6)Fuc(1)NeuAc(2)<br>QDQCIYNTTYLNVQR-HexNAc(6)Hex(7)Fuc(1)NeuAc(2)<br>QDQCIYNTTYLNVQR-HexNAc(6)Hex(7)NeuAc(1)<br>QDQCIYNTTYLNVQR-HexNAc(6)Hex(7)NeuAc(2)<br>QDQCIYNTTYLNVQR-HexNAc(6)Hex(7)NeuAc(3)<br>QNQCFYNSSYLNVQR-HexNAc(6)Hex(7)NeuAc(1)<br>QNQCFYNSSYLNVQR-HexNAc(6)Hex(7)NeuAc(2)<br>QNQCFYNSSYLNVQR-HexNAc(6)Hex(7)NeuAc(3)<br>QNQCFYNSSYLNVQR-HexNAc(6)Hex(7)NeuAc(4)	LGNWSAMPSCK-HexNAc(4)Hex(5)NeuAc(2)<br>QDQCIYNTTYLNVQR-HexNAc(6)Hex(7)Fuc(1)NeuAc(3)<br>QNQCFYNSSYLNVQR-HexNAc(6)Hex(7)Fuc(1)NeuAc(4)	AANGSLR-HexNAc(2)Hex(9)<br>CGLVPVLAENYNK-HexNAc(5)Hex(6)NeuAc(2)<br>ENGTISR-HexNAc(5)Hex(6)Fuc(1)NeuAc(1)<br>ENGTISR-HexNAc(8)Hex(9)Fuc(1)NeuAc(4)<br>LLNINPNK-HexNAc(4)Hex(5)Fuc(1)<br>NNHTASILDR-HexNAc(3)Hex(4)Fuc(1)<br>QQQHLFGSNVTDCSGNFCLFR-HexNAc(4)Hex(5)Fuc(1)NeuAc(2)<br>QQQHLFGSNVTDCSGNFCLFR-HexNAc(5)Hex(6)Fuc(1)NeuAc(3)<br>SVQEIQATFFYFTPNK-HexNAc(5)Hex(6)NeuAc(3)<br>SVQEIQATFFYFTPNKTEDTIFLR-HexNAc(4)Hex(5)NeuAc(2)<br>SVQEIQATFFYFTPNKTEDTIFLR-HexNAc(5)Hex(6)NeuAc(2)<br>SVQEIQATFFYFTPNKTEDTIFLR-HexNAc(5)Hex(6)NeuAc(3)<br>SVQEIQATFFYFTPNKTEDTIFLR-HexNAc(6)Hex(7)NeuAc(4)<br>VSNYSR-HexNAc(4)Hex(5)Fuc(1)NeuAc(2)<br>VVHAVEVALATFNAESNGSYLQLVEISR-HexNAc(5)Hex(6)NeuAc(2)<br>WFYIASAFRNEEYNK-HexNAc(5)Hex(6)Fuc(1)NeuAc(3)<br>YHYNGTFEDGK-HexNAc(2)Hex(6)	-	AANGSLR-HexNAc(2)Hex(8)<br>ALPQPQNVTSLLGCTH-HexNAc(4)Hex(5)NeuAc(2)<br>CGLVPVLAENYNK-HexNAc(4)Hex(5)NeuAc(1)<br>CGLVPVLAENYNK-HexNAc(4)Hex(5)NeuAc(2)<br>CGLVPVLAENYNK-HexNAc(5)Hex(6)NeuAc(1)<br>EEQFNSTFR-HexNAc(4)Hex(5)Fuc(1)<br>ENGTISR-HexNAc(4)Hex(5)NeuAc(1)<br>ENGTISR-HexNAc(4)Hex(5)NeuAc(2)<br>ENGTISR-HexNAc(5)Hex(6)Fuc(1)NeuAc(2)<br>ENGTISR-HexNAc(5)Hex(6)Fuc(1)NeuAc(3)<br>ENGTISR-HexNAc(5)Hex(6)NeuAc(1)<br>ENGTISR-HexNAc(5)Hex(6)NeuAc(2)<br>ENGTISR-HexNAc(5)Hex(6)NeuAc(3)<br>ENGTISR-HexNAc(6)Hex(7)Fuc(1)NeuAc(3)<br>ENGTISR-HexNAc(6)Hex(7)Fuc(1)NeuAc(4)<br>ENGTISR-HexNAc(6)Hex(7)NeuAc(4)<br>ENGTISR-HexNAc(7)Hex(8)Fuc(1)NeuAc(4)<br>FSDGLESNSSTQFEVK-HexNAc(2)Hex(9)<br>GHTLTLNFTR-HexNAc(2)Hex(6)<br>GHTLTLNFTR-HexNAc(2)Hex(7)<br>GSQWSDIEEFCNR-HexNAc(4)Hex(5)NeuAc(2)<br>HLNGTITAK-HexNAc(2)Hex(8)<br>KLCPDCPLLAPLNDSR-HexNAc(4)Hex(5)NeuAc(2)<br>KLCPDCPLLAPLNDSR-HexNAc(5)Hex(6)NeuAc(2)<br>KLCPDCPLLAPLNDSR-HexNAc(5)Hex(6)NeuAc(3)<br>LCPDCPLLAPLNDSR-HexNAc(4)Hex(5)NeuAc(1)<br>LCPDCPLLAPLNDSR-HexNAc(4)Hex(5)NeuAc(2)<br>LCPDCPLLAPLNDSR-HexNAc(5)Hex(6)NeuAc(2)<br>LCPDCPLLAPLNDSR-HexNAc(5)Hex(6)NeuAc(3)<br>LLNINPNK-HexNAc(5)Hex(6)Fuc(1)<br>MNFTGGDTCHK-HexNAc(2)Hex(8)<br>NACCSTNTSQEAHK-HexNAc(4)Hex(5)Fuc(1)NeuAc(2)<br>NATLAEQAK-HexNAc(2)Hex(8)<br>NEEYNK-HexNAc(4)Hex(5)NeuAc(2)<br>NEEYNK-HexNAc(5)Hex(6)NeuAc(2)<br>NEEYNK-HexNAc(5)Hex(6)NeuAc(3)<br>NYTADYDK-HexNAc(2)Hex(8)<br>QDQCIYNTTYLNVQR-HexNAc(4)Hex(5)NeuAc(2)<br>QDQCIYNTTYLNVQR-HexNAc(5)Hex(6)Fuc(1)NeuAc(3)<br>QDQCIYNTTYLNVQR-HexNAc(5)Hex(6)NeuAc(2)<br>QDQCIYNTTYLNVQR-HexNAc(5)Hex(6)NeuAc(3)<br>QDQCIYNTTYLNVQR-HexNAc(6)Hex(7)Fuc(1)NeuAc(4)<br>QDQCIYNTTYLNVQR-HexNAc(6)Hex(7)NeuAc(4)<br>QNQCFYNSSYLNVQR-HexNAc(5)Hex(6)Fuc(1)NeuAc(3)<br>QNQCFYNSSYLNVQR-HexNAc(5)Hex(6)NeuAc(2)<br>QNQCFYNSSYLNVQR-HexNAc(5)Hex(6)NeuAc(3)<br>QQQHLFGSNVTDCSGNFCLFR-HexNAc(4)Hex(5)NeuAc(2)<br>QQQHLFGSNVTDCSGNFCLFR-HexNAc(5)Hex(6)NeuAc(3)<br>RPTGEVYDIEIDTLETTCHVLDPTPLANCSVR-HexNAc(4)Hex(5)NeuAc(2)<br>RPTGEVYDIEIDTLETTCHVLDPTPLANCSVR-HexNAc(5)Hex(6)NeuAc(2)<br>RPTGEVYDIEIDTLETTCHVLDPTPLANCSVR-HexNAc(5)Hex(6)NeuAc(3)<br>VVHAVEVALATFNAESNGSYLQLVEISR-HexNAc(5)Hex(6)NeuAc(3)<br>WFYIASAFRNEEYNK-HexNAc(4)Hex(5)NeuAc(2)<br>WFYIASAFRNEEYNK-HexNAc(5)Hex(6)NeuAc(3)<br>YHYNGTLLDGTSFDTSYSK-HexNAc(2)Hex(8)<br>YIHQNYTK-HexNAc(2)Hex(8)

Fig. S3: List of *N*-glycopeptide Structures Corresponding to the Overlap of Search Engine Identifications. Unique *N*-glycopeptide structure list for upset plot results: a combined hit list for the four search engines, detailing which unique *N*-glycopeptide structures were identified by which search engines or search engine ensembles during the energy-dependent studies.

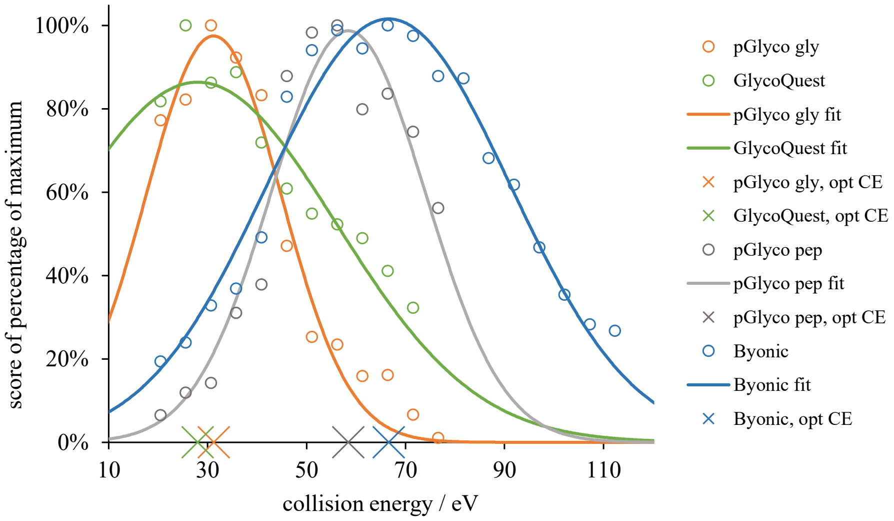

Fig. S4 Score vs. CE Curves for QDQCIYNTTYLNVQR-HexNAc(5)Hex(6)NeuAc(2)$^{4+}$. Experimental points together with the fitted Gaussian functions for all investigated identification scores for the example peptide QDQCIYNTTYLNVQR-HexNAc(5)Hex(6)NeuAc(2)$^{4+}$. Symbols denote measured data, while solid lines depict the model functions. The peak positions of the latter are marked by crosses on the horizontal axis. Byonic; Grey: pGlyco peptide; Orange: pGlyco glycan; Green: GlycoQuest.

Correlation between Retention Time and Peptide Backbone Hydrophobicity

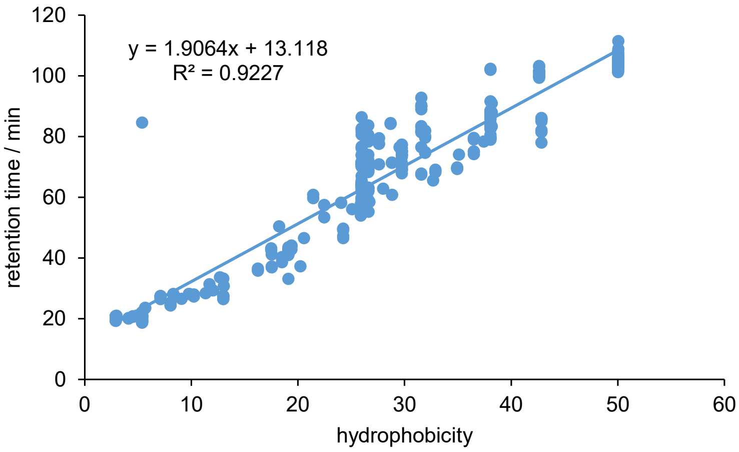

Fig. S5 Retention time of *N*-glycopeptides vs. hydrophobicity of the peptide backbone for the investigated *N*-glycopeptides. We determined hydrophobicity values using the peptide analyzing tool of Thermo Fisher Scientific (Thermo Fisher Scientific. Peptide Synthesis and Proteotypic Peptide Analyzing Tool https://www.thermofisher.com/hu/en/home/life-science/protein-biology/peptides-proteins/custom-peptide-synthesis-services/peptide-analyzing-tool.html.)

Charge Dependence of the Optimal CE for the Various Search Engine Scores

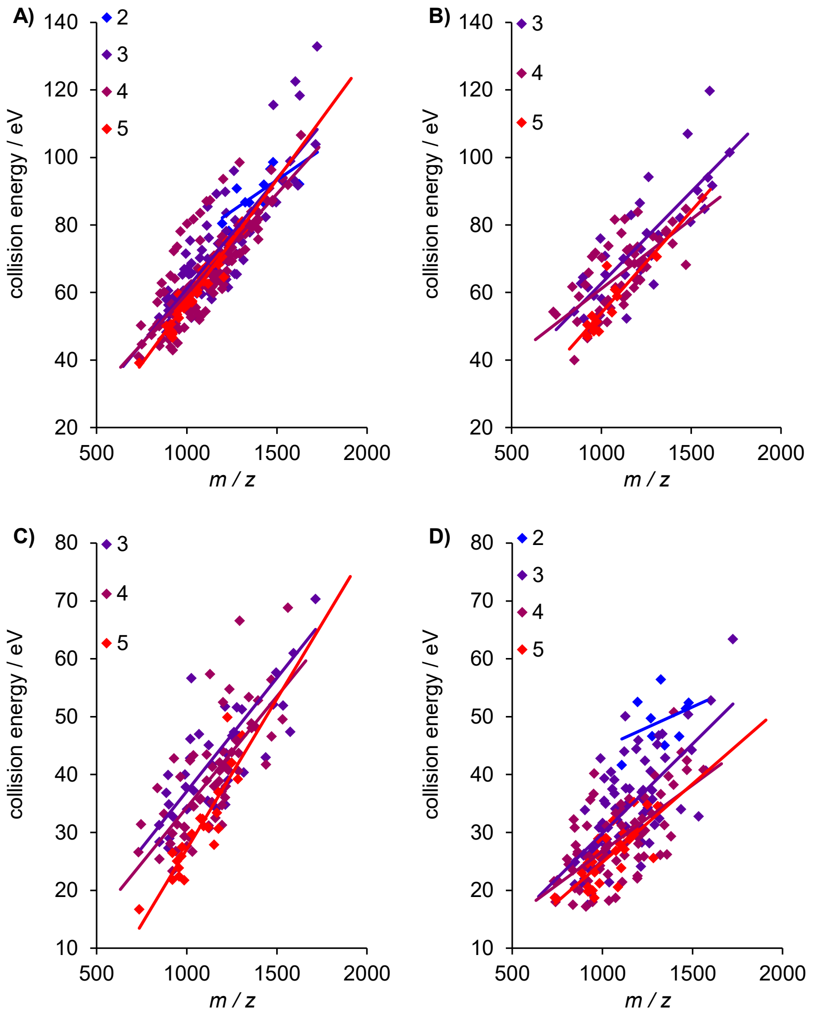

Fig. S6 Optimal collision energies of *N*-glycopeptides in eV as a function of *m/z* for the various search engine scores and their dependence on the charge state. A) Byonic, B) pGlyco peptide, C) pGlyco glycan, D) GlycoQuest

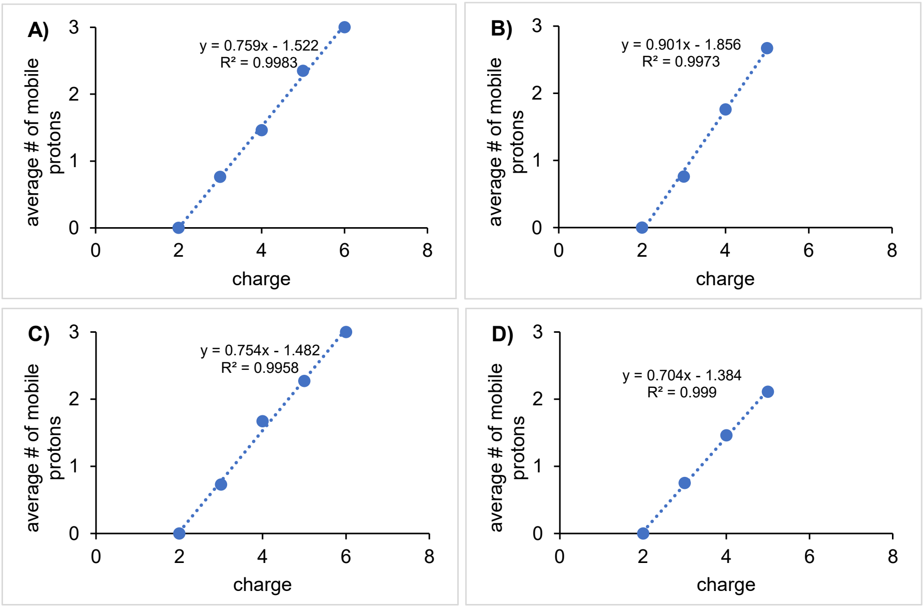

Fig. S7 The average number of mobile protons as a function of charge state. A) Byonic, B) pGlyco peptide, C) pGlyco glycan, D) GlycoQuest

Effect of Number of SA Units on the Optimal CE for the Various Search Engine Scores

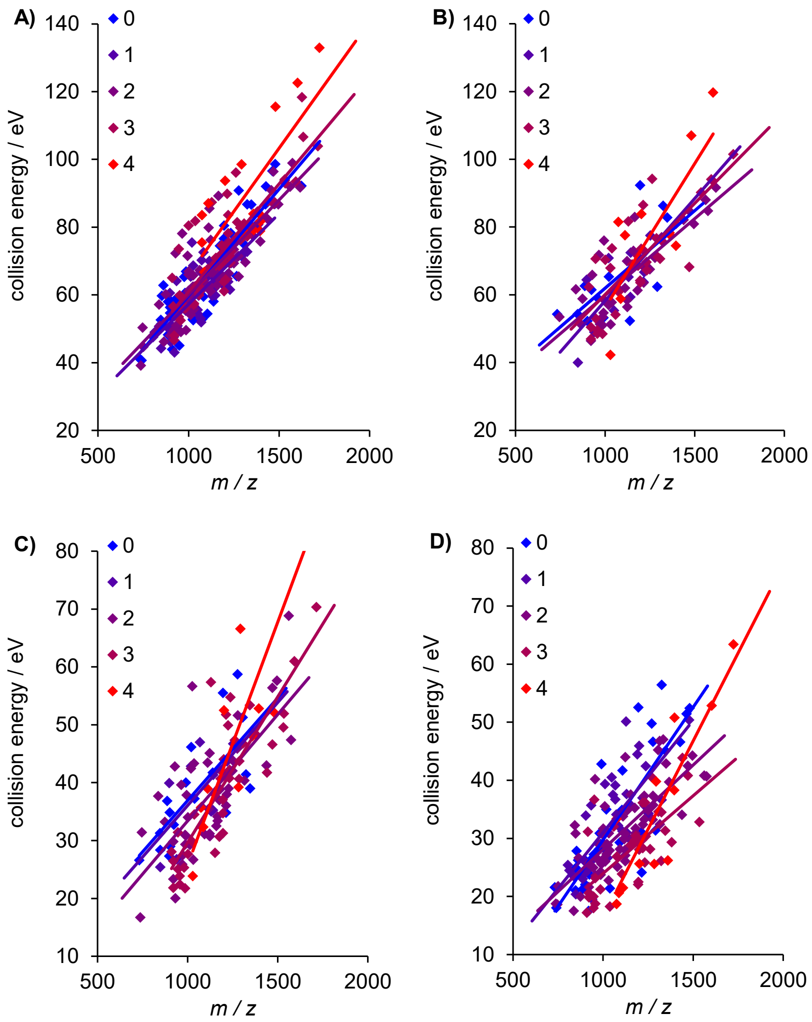

Fig. S8 Optimal collision energies of *N*-glycopeptides in eV as a function of *m/z* for the various search engine scores and their dependence on the number of sialic acid (SA) units. A) Byonic, B) pGlyco peptide, C) pGlyco glycan, D) GlycoQuest

Effect of Sample Complexity

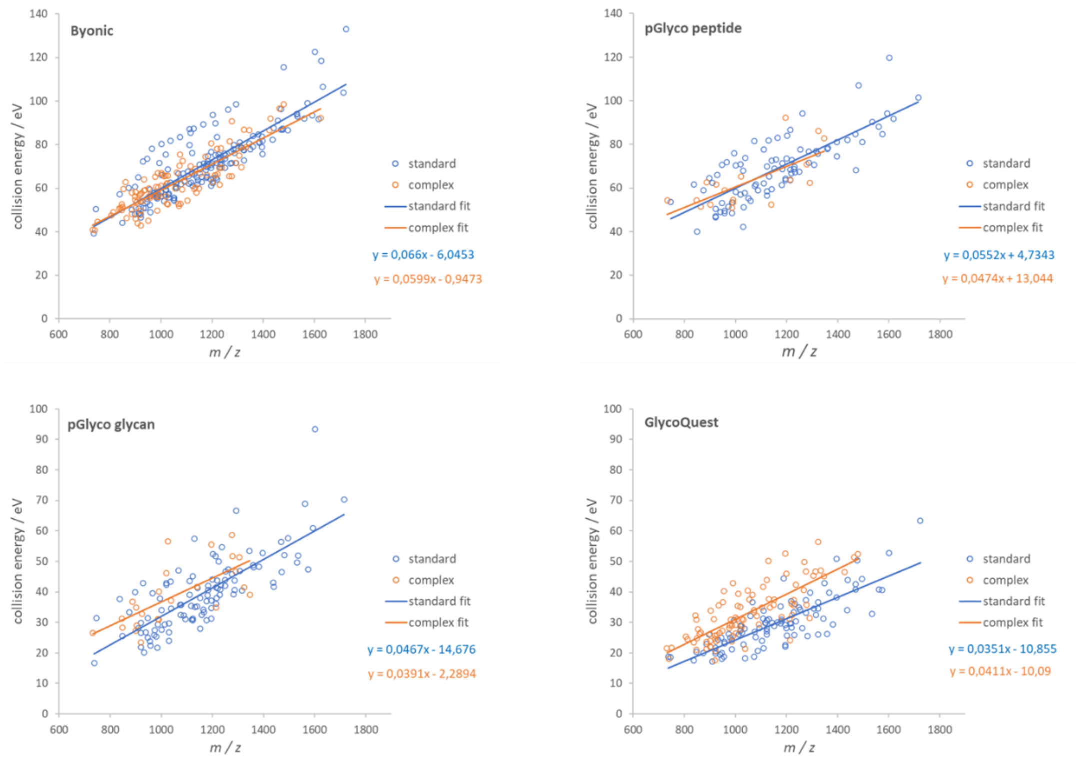

Fig. S9. Optimal Collision Energies of *N*-glycopeptides for Standards and Complex Samples. Collision energy values are presented in eV as a function of *m/z* for various search engine scores for standard (AGP, fetuin and transferrin – blue) and complex (HeLa and plasma – orange) samples. Circles indicate the experimental points while lines represent linear fits of the measured data.

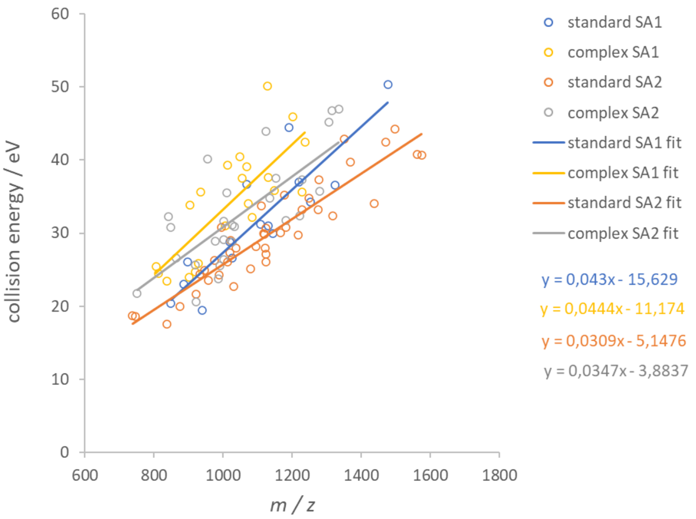

Fig. S10. Optimal Collision Energies of *N*-glycopeptides for GlycoQuest Search Engine for Standards and Complex Samples with 1 or 2 Sialic Acid Units. Collision energy values are presented in eV as a function of *m/z*. Blue and orange: standards (AGP, fetuin and transferrin); Yellow and grey: complex samples (HeLa and plasma). Circles indicate the experimental points while lines represent linear fits of the measured data.

Results from Lasso Regression

Table S6. R^2 and root mean squared error (in eV) of various Lasso regression models applied to optimal CEs for various search engine scores, as well as a few unregularized models for reference. For Lasso regression, the cells also show the independent variables that enter with nonzero coefficients as compared to the model with the next higher α value.

Model	Byonic	pGlyco peptide score	pGlyco glycan score	GlycoQuest
**Unregularized; independent variables: *m*/*z*, peptide *m*/*z*, total mass, glycan mass, hydrophobicity**	89.29% / 4.920	87.35% / 5.092	68.43% / 6.448	64.66% / 5.250
**Unregularized, including all independent variables**	95.99% / 3.012	96.09% / 2.833	82.98% / 4.734	74.27% / 4.479
**α=1**	91.32% / 4.430<br>+ number of F,N,T,Q	91.25 % / 4.237<br>+ isoelectric point	73.60% / 5.896<br>+ isoelectric point<br>+ number of Q	65.45% / 5.191<br>+ position of glycan<br>+ isoelectric point
**α=3**	89.54 % / 4.863<br>+ position of glycan	89.02% / 4.744	68.85% / 6.404<br>+ total mass<br>+ position of glycan	64.34% / 5.273<br>+ hydrophobicity
**α=5**	88.94 % / 5.001	87.74% / 5.012<br>+ position of glycan<br>+ hydrophobicity<br>+ total mass	67.86% / 6.505<br>– total mass<br>+ hydrophobicity	64.02% / 5.297
**α=10**	87.89% / 5.234<br>+ hydrophobicity	86.54 % / 5.254<br>+ glycan mass<br>– total mass	67.40% / 6.552	63.67% / 5.322
**α=20**	87.13 % / 5.395<br>+ peptide *m*/*z*	86.21 % / 5.317<br>+ peptide *m*/*z*<br>– glycan mass	67.26% / 6.566<br>+ peptide *m*/*z*	62.96% / 5.374<br>+ peptide *m*/*z*
**α=100**	85.38% / 5.750<br>Nonzero coefficients: *m*/*z*, glycan mass, total mass	84.33 % / 5.669<br>Nonzero coefficients: *m*/*z*, glycan mass, total mass	66.39% / 6.653<br>Nonzero coefficients: *m*/*z*, glycan mass, total mass	60.98% / 5.516<br>Nonzero coefficients: *m*/*z*, glycan mass, total mass
**Unregularized; only *m*/*z* as independent variable**	74.69% / 7.565	60.92% / 8.952	56.13% / 7.601	48.28% / 6.351
